# 分子印迹技术在催化领域应用研究进展

**DOI:** 10.3724/SP.J.1123.2025.05019

**Published:** 2026-01-08

**Authors:** Weimin YE, Dongcheng HE, Xinjiang CUI, Haowen MA, Bo QIAN, Feng SHI

**Affiliations:** 1. 中国科学院兰州化学物理研究所，低碳催化与二氧化碳利用全国重点实验室，甘肃 兰州 730000; 1. State Key Laboratory of Low Carbon Catalysis and Carbon Dioxide Utilization，Lanzhou Institute of Chemical Physics，Chinese Academy of Sciences，Lanzhou 730000，China; 2. 中国科学院大学，北京 100049; 2. University of Chinese Academy of Sciences，Beijing 100049，China; 3. 中国石油石油化工研究院，兰州化工研究中心，甘肃 兰州 730000; 3. Lanzhou Petrochemical Research Center，Petrochemical Research Institute，PetroChina Company Limited，Lanzhou 730000，China

**Keywords:** 分子印迹技术, 分子印迹催化剂, 催化反应, 研究进展, molecular imprinting technology, molecularly imprinted catalyst, catalytic reaction, research progress

## Abstract

在催化反应中，催化剂活性和选择性的提高不仅能够增加目标产物的产率，还可在减少反应过程复杂程度的同时节约反应能耗、降低副产物的生成。分子印迹技术（MIT）作为一种优异的催化剂制备技术，可用于制备具有高活性、高选择性和热稳定性的分子印迹催化剂（MIC），能够有效解决上述问题。分子印迹催化剂结合生物酶催化的原理，在分子印迹催化剂中构筑具有特定催化活性位点及空间构型的分子印迹空穴，赋予其优异的分子识别能力，充分利用可逆共价相互作用、静电引力、氢键等相互作用筛分反应底物、反应中间体以及反应产物的结构和官能团，以实现特定的反应过程并高选择性地得到目标产物。本文主要综述了分子印迹技术在热催化领域中的相关研究，阐述了分子印迹技术的基本原理、相关理论及其发展历程，介绍了本体聚合、液相悬浮聚合、沉淀聚合和表面分子印迹等典型的分子印迹催化剂合成方法、分子印迹催化剂的结构表征技术（傅里叶变换红外光谱、有机元素组成分析、高分辨质谱仪等），随后重点展示了分子印迹催化剂（包括贵金属、非贵金属、无金属催化剂等）在水解反应、氧化反应、还原反应、偶联反应、聚合物反应器等催化反应中的研究进展，还简要陈述了分子印迹技术在光/电催化、人工酶催化以及传感器、吸附分离等其他领域的应用，最后总结了分子印迹技术在催化领域应用中存在的若干问题并展望了其未来发展趋势。

## Abstract (en)

The synergistic enhancement of catalytic activity and selectivity constitutes a critical challenge in modern heterogeneous catalysis， which directly influences target product yield， reaction energy efficiency， and process economics. Molecular imprinting technology （MIT） has demonstrated exceptional potential in overcoming this limitation by enabling the rational design of molecularly imprinted catalysts （MICs） with high activity， superior selectivity， and favorable thermal stability. These advanced catalysts combine biomimetic recognition with heterogeneous catalysis， wherein precisely engineered imprinted cavities integrate two key structural features， including catalytically active sites with tailored electronic properties and molecular imprinting cavities with specific structure. These imprinted cavities endow MIC exhibit exceptional molecular recognition capabilities， enabling selective binding to substrates， intermediates， and products via reversible covalent bonds， electrostatic interactions， hydrogen bonding， and other noncovalent forces. This precise recognition facilitates the mediation of specific reaction pathways， ensuring high-selectivity synthesis of target compounds. The preparation of MIC typically involves three sequential steps： template molecule assembly， template configuration fixation， and template molecule elution. In the template assembly stage， reversible interactions are commonly employed to drive the self-assembly of template molecules （target-structured molecules） with functional monomers， forming stable imprinted complexes. For template configuration fixation， cross-linking polymerization or surface engineering techniques are predominantly utilized to immobilize the assembled structure， ensuring the preservation of cavity geometry after template removal. Subsequent elution of the template molecules generates imprinted cavities on the MIC surface. By optimizing template assembly methodologies and fixation strategies based on application-specific requirements， both the cavity structure and catalytic binding modes can be precisely modulated， thereby enhancing catalytic activity and selectivity for tailored catalyst design. Additionally， the introduction of precious metals （e.g.， Rh， Ru， Au， Ag） and non-precious metals （e.g.， Fe） as catalytic active sites further augments MIC performance. Despite the promising application potential of MICs in chemical synthesis， their preparation and characterization remain challenged by several key limitations， like the sub-nanostructured imprinted cavities hindering detailed structural elucidation of binding sites， template molecules encapsulation within the polymer matrix during cross-linking resulting in incomplete elution， quantitative analysis of metal species in polymer-based MICs lacking standardized methodologies. To address these challenges and guide design of high-performance MICs， researchers have integrated advanced characterization techniques to comprehensively evaluate MIC structure， including morphology， elemental composition， active site distribution， chemical bonding information， and metal coordination environments. Currently， MICs exhibit tremendous application potential in the synthesis of various fine chemical products， but related review articles focusing on MIC are relatively scarce. This review focuses on the applications of MIT in thermal catalysis， systematically discussing its fundamental principles， theoretical foundations， and historical development. Next， various typical synthetic strategies for MICs， including bulk， suspension， precipitation， and surface imprinting polymerization are summarized. Then series of key characterization methods， such as Fourier transform infrared spectroscopy （FT-IR）， elemental analysis （EA）， and high-resolution mass spectrometry （HRMS） are described to analyze the structure of MICs. Moreover， different types of MICs （noble metal， non-noble metal， and metal free MICs） are used in catalytic reactions， including hydrolysis， oxidation， reduction， coupling， and polymerization. In addition， the photo-/electrocatalysis， artificial enzyme design， sensing， and adsorption/separation are also discussed as emerging applications of MIT. Finally， the research challenges and future directions are proposed in this field.

分子印迹技术（molecular imprinting technology， MIT）是一类模拟生物酶-底物相互作用对印迹分子（模板分子）实现专一性识别的技术。生物酶因其在温和条件下对反应底物具有极高的催化活性和特异性识别能力^［1］^而受到广泛关注。然而，生物酶在强酸强碱、高温高压等条件下无法有效发挥催化作用，使其难以广泛应用于复杂环境的化学反应过程^［2，3］^。因此，在研究生物酶结构和特点的基础上，通过仿生策略合成类生物酶结构的催化材料来模拟酶催化反应过程，开发出具有酶促反应优点且能耐受一定温度、压力和酸碱性的催化材料及其催化反应技术具有重要意义^［4-6］^。

Dickey等参考了酶促反应的特点和人造抗体的理念，于1949年首次提出了分子印迹的概念^［7］^，Wulff等^［8］^在1972年首次合成了分子印迹聚合物。自1990年以来，在特异性识别的基础上逐步发展出了分子印迹技术，并广泛应用于有机催化领域的催化材料制备和不对称合成反应等。例如，通过构筑类抗原-抗体系统或类酶催化体系，可以制备出具有分子识别能力且具有优异稳定性和循环使用性能的分子印迹催化剂（molecularly imprinted catalyst， MIC）^［9，10］^；此外，在有机不对称合成反应中，利用MIT合成的MIC展现出远高于传统催化剂的不对称催化性能，具有极高的产物构型选择性^［11，12］^。如今，MIT已被广泛应用于化学传感器^［13］^、分离富集^［14，15］^、有机催化^［16，17］^、光/电催化^［18，19］^等领域。

MIT的相关理论是在Fischer的“锁钥模型”、Pauling的抗体形成学说和Dickeyde的“专一性吸附理论”等基础上逐渐形成的^［20-22］^。在分子印迹形成过程中，反应底物、反应过渡态和目标产物及其类似物均可作为分子印迹模板。图1a展示了MIT的简单过程，模板分子通过共价或非共价相互作用与功能单体形成印迹复合物，在交联剂的作用下进一步固定构型，除去模板分子后形成具有特定空间结构的分子印迹空穴。只有与分子印迹空穴相匹配的反应物分子才能与之反应形成特定结构的产物，从而实现催化的高效性和专一性。

**图1:**
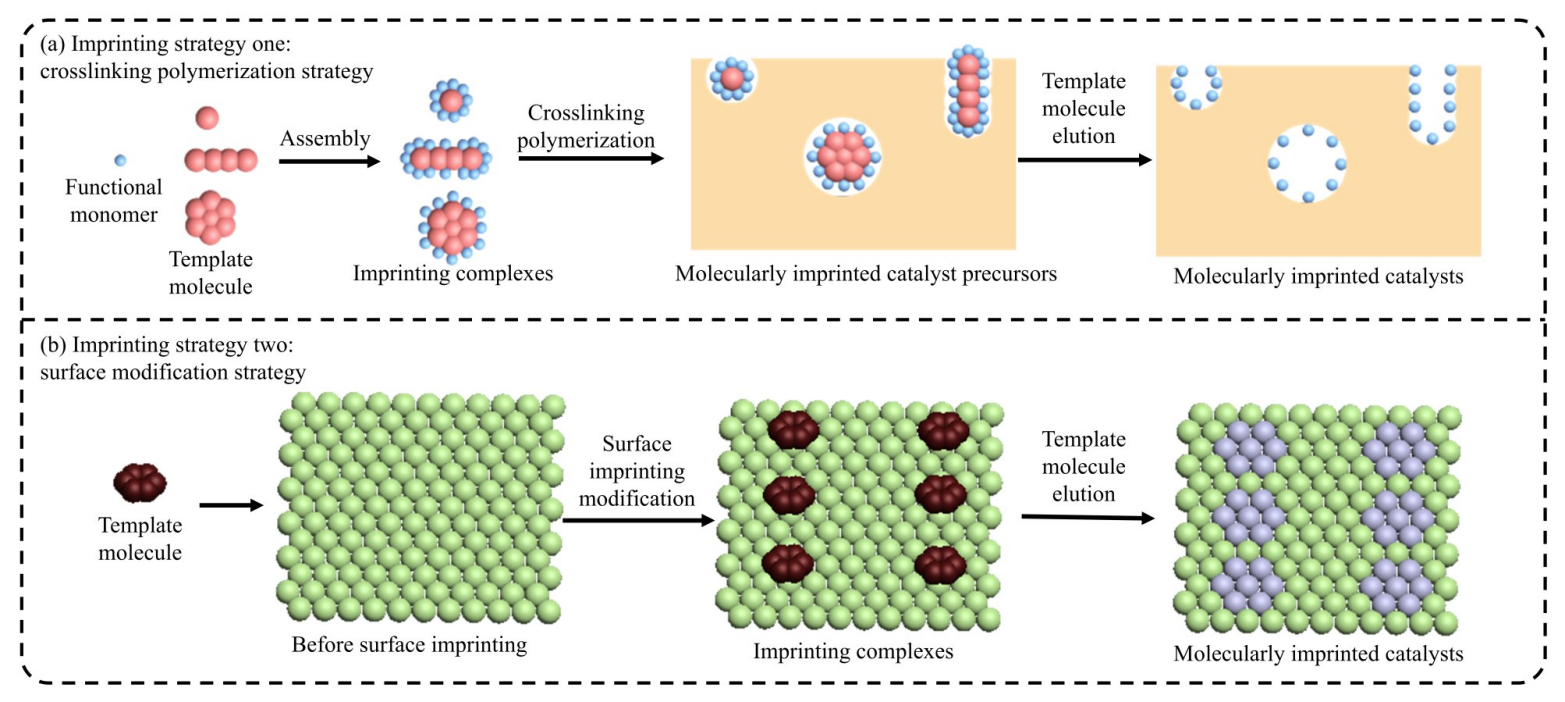
分子印迹技术原理示意图

当前，通过MIT合成的MIC已被应用于水解反应、氧化反应、还原反应、C-C/C-N偶联反应等化学反应。此外，相比于生物酶催化剂，MIC具有良好的催化稳定性和反应条件耐受性（较高的温度、压力和一定范围的酸碱性），是一种理想的有机化学催化剂。MIT技术与前沿高分子材料制备技术相结合还可以构筑具有自响应、自修复、优异级联催化性能的新型分子印迹聚合物反应器，实现对产物的高选择性合成和对多步化学反应的智能控制。近年来，MIT技术已发展为一种有效的表面修饰技术：通过MIT可以在载体表面构筑具有分子识别能力的特异性催化活性位点，赋予载体特异性催化反应活性^［23，24］^（图1b）。

为了更好地了解MIT的发展历程及其在催化领域的应用，本文系统综述了该领域的相关文献。概述了MIT的基本概念和常见制备方法（包括常用的功能单体和交联剂、聚合印迹策略和表面印迹策略等）；介绍了在MIC设计、制备和表征过程中应用的典型表征技术，并讨论了各类表征技术在MIC表征过程中的优缺点；重点阐述了MIC在热催化领域的应用，并分类讨论了MIC的典型反应场景和应用现状，主要包括MIT在水解反应、贵金属/非贵金属以及无金属催化的氧化反应、手性还原反应、非手性还原反应、C-C偶联反应、C-N偶联反应和聚合物反应器中的应用；还展示了MIT在部分重要的光/电催化反应和酶催化反应研究中的具体应用。最后，在分析MIT在催化领域研究现状的基础上，提出了现有MIT发展中存在的若干问题，并对其未来发展趋势提出了展望。

## 1 分子印迹技术及其应用

MIT是一系列用于制备分子印迹功能材料的技术统称。基于MIT的基本原理，在制备过程中，根据模板分子的特点，选用与其结构互补的功能分子进行分子印迹过程，由此获得的材料对特定分子具有优异的分子识别能力。在催化领域中，常通过MIT制备高选择性的分子印迹催化剂（MIC）。MIC表现出的优异选择性可以大幅提高反应效率，简化制备、分离、提纯等过程，在热催化、光/电催化和酶催化中都有重要应用。

### 1.1 分子印迹催化剂

MIC是通过MIT制备的一种催化材料，由于这类催化剂大多数以聚合物的形式存在，狭义上又用分子印迹聚合物（MIP）指代MIC。MIC表面存在大量与模板分子空间结构和官能团匹配的空穴和负载位点，可精准识别模板分子并催化特定化学反应。根据模板分子种类的差异，MIC可以大致分为底物分子印迹聚合物（S-MIC）、反应过渡态类似物分子印迹聚合物（TSA-MIC）和目标产物分子印迹聚合物（P-MIC）。由于S-MIC和P-MIC具有优异的择形性，在化学反应过程中，只有与MIC空穴空间结构完全匹配的底物或产物才可以自由进出空穴，从而催化特定化学反应；对于TSA-MIC，在催化过程中，聚合物空穴的空间结构引导底物形成与之结构匹配的反应过渡态，同时空穴中的特定位点与底物结合稳定过渡态结构，促进了目标产物的生成^［25］^。

### 1.2 分子印迹催化剂的制备

MIC的制备大致分为混合组装、印迹形成、模板去除3个步骤，具体过程为：（1）混合后的模板分子和功能单体（功能配体）通过共价或非共价相互作用（如：静电引力、氢键、金属螯合、范德华力等^［26-28］^）自组装，形成印迹复合物；（2）印迹复合物在交联剂作用下被固定在聚合物表面，形成刚性分子骨架和分子印迹空穴；（3）通过溶剂洗脱、反应脱附等方法除去骨架/空腔中的模板分子，形成具有优异择形性和特异性分子结合位点的分子印迹空穴，得到具有高选择性的分子印迹催化材料（即MIC）。

MIC的制备过程主要涉及模板分子、功能单体（功能配体）、交联剂3类化学物质，其中功能单体和交联剂的选用是MIC制备的关键。根据文献报道统计，常用的功能单体（功能配体）有丙烯酰胺（AM）及其衍生物（如2-丙烯酰胺-2-甲基丙磺酸（AMPS）^［29，30］^、*N，N*-二甲基丙烯酰胺^［31］^、*N，N*-二乙基丙烯酰胺等^［32］^）、4-乙烯基吡啶（4-VPy）^［33］^、甲基丙烯酸（MAA）^［34，35］^等（结构式见图2a），这类有机分子通常含有富电子的含氮官能团或强亲核性的含氧官能团，可以通过静电引力、氢键等相互作用与模板分子官能团自发相连，形成空间结构相对固定的印迹复合物。此外，功能单体的分子中通常含有不饱和碳链，可以在后续制备过程中与交联剂相互作用形成具有特定空间结构的刚性骨架。交联剂是液相中印迹复合物间相互连接的桥梁，通常具有不饱和碳链，可以与功能单体发生交联聚合反应，稳定印迹复合物的空间结构。MIC合成中常用的交联剂为乙二醇二甲基丙烯酸酯（EGDMA）^［12］^、*N，N′*-亚甲基双丙烯酰胺（MBA）^［29，36］^和二乙烯基苯（DVB）^［37，38］^等，结构式如图2b所示。

**图2:**
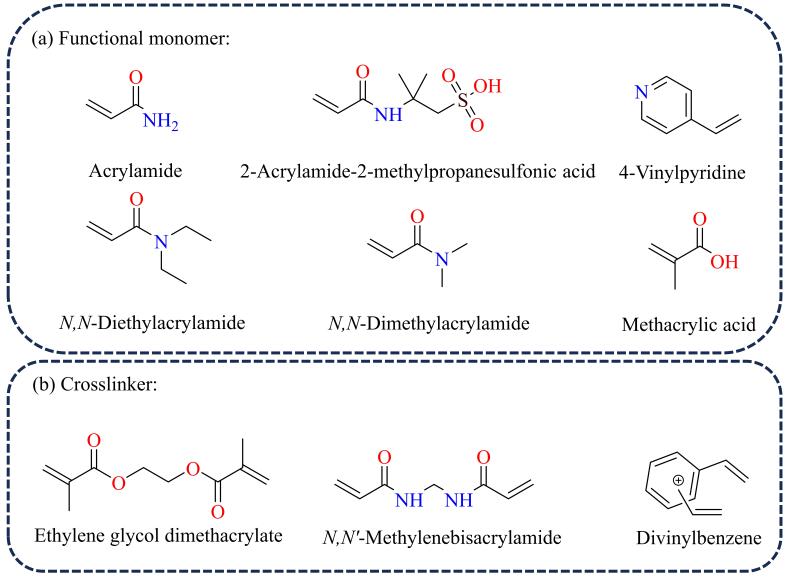
分子印迹催化剂合成中常用的功能单体和交联剂

交联剂在分子印迹过程中也发挥着重要作用，通过选择不同种类的交联剂，可以调控所得MIC的催化活性。例如，在MIC制备过程中，结合能力较强的交联剂可以获得更加稳定的分子印迹空穴，使催化剂的比表面积更高，有利于目标分子的特异性吸附。但是，目标分子在分子印迹空穴和非印迹框架中存在竞争吸附，导致特异性吸附效果减弱。此外，交联度更高的印迹空穴会造成印迹位点包埋过深，降低MIC的分子识别能力。

在追求MIC优异催化性能的过程中，MIC制备技术也不断更新，发展出了本体聚合法、液相悬浮聚合法、沉淀聚合法和表面印迹法等适用于不同催化反应或应用条件的MIC制备技术。

本体聚合 本体聚合是一种传统的MIC合成策略，该策略在MIC制备过程中，功能单体在非共价相互作用下排列在印迹分子周围，与交联剂聚合后形成体积较大的聚合物，随后经历研磨破碎、筛分、洗脱印迹分子后，得到尺寸适宜的MIC。Severin团队^［12］^通过本体聚合法，将Ru（Ⅱ）与具有一个或两个苯乙烯侧链的*N*-磺酰基-1，2-乙二胺配体配位，制备Ru（Ⅱ）Cl-（η^6^-烯烃）功能单体。随后Rh（Ⅱ）功能单体在非共价相互作用下与膦配体配位并与EGDMA聚合后形成分子印迹，得到的聚合物经过研磨破碎、筛分、洗脱膦配体后，制得可催化二苯甲酮还原反应的MIC。本体聚合法实现了MIC的高效、简便制备，是一种普遍使用的MIC制备方法。然而，该MIC制备策略中涉及费时耗能的研磨、筛分工序，且该工序容易导致MIC内部微观结构破碎、聚合物颗粒尺寸不均一、材料的理化性质再现性较差、催化性能重复性差等问题，因此难以通过该策略实现MIC的批量化制备^［39］^。

液相悬浮聚合 液相悬浮聚合是一种常见的球形MIC制备策略，具有MIC尺寸可调、制备时间短、能耗低等优点。溶剂极性是液相悬浮聚合制备优异分子识别性能MIC的关键，若液相溶剂与功能单体和/或模板分子的极性差距过大，功能单体将难以与足量的模板分子相互作用，从而降低模板分子与功能单体之间相互作用的强度，大幅降低MIC的分子识别能力^［40］^。此外，在液相悬浮聚合制备MIC的过程中，模板分子用量和混合强度对MIC粒径有显著影响。使用强极性分子制备MIC时，若模板分子用量过高且混合强度过大，将易发生模板分子团聚现象，不利于小尺寸MIC的制备^［41］^。

沉淀聚合 沉淀聚合法是一种合成具有较大比表面积和优异催化活性的纳米/微米级球形MIC的有效策略，在催化领域中具有广阔应用前景。在分子印迹微球催化剂制备过程中，须控制模板分子、交联剂和功能单体浓度，以形成低浓度混合溶液，从而通过可控的小范围聚合反应制备分子印迹微球。若所需MIC微球粒径较小，可以在沉淀聚合过程中加入胶体稳定剂（如：全氟表面活性剂等），以显著降低所得MIC微球的尺寸^［41］^。此外，沉淀聚合策略也可以实现固体材料表面改性，如Guo等^［33］^通过沉淀聚合策略向含有乙烯基的多壁碳纳米管（MWCNT）表面接枝MIC改性，成功制备出可催化硝基苯磷酸酯水解并对水解产物具有吸附性能的MWCNT-MIC复合材料。

表面分子印迹技术 印迹分子的洗脱是MIC合成过程中的关键步骤之一，分子洗脱率与MIC的合成效率相关，较低的印迹分子洗脱率将降低MIC的合成效率。表面分子印迹技术是一种理想的MIC合成策略，通过对催化剂载体进行表面分子印迹改性，可以高效制备具有优异选择性的催化剂^［42］^。该印迹策略克服了MIC聚合过程中因印迹分子包埋过深而难以洗脱的缺点，且拓展了分子印迹技术的应用范围。Ordomsky和Khodakov等^［16］^提出了一种新型表面分子印迹策略，通过在钯表面依次吸附芳香族模板剂和催化剂毒性剂，定向毒害部分活性位点，成功制备出一种具有分子识别催化活性的MIC。该MIC具有优异的底物识别能力，可以高效催化与模板分子结构相近的底物反应。然而，通过表面分子印迹技术定向毒害部分催化活性位点虽然可以制备出具有较高催化选择性的催化剂，但是在制备过程中容易造成催化剂失活，且牺牲了大量催化剂活性位点。因此Shi和Cui等^［17］^以无硝基芳香化合物加氢还原催化活性的Cu/Al_2_O_3_为载体，硝基芳香化合物为印迹分子，1，10-菲啰啉为功能单体，成功制备出可以催化特定硝基芳香化合物加氢还原的Cu-MIC。

其他印迹策略 除了上述4种典型分子印迹策略之外，根据特定应用需求及所需MIC的特点，还可以通过溶胶-凝胶法、电化学聚合法等印迹策略制备出具有不同理化性质的MIC^［43-45］^。通过溶胶-凝胶印迹策略，可以将分子模板引入无机网络结构中，制得兼有溶胶-凝胶和分子印迹材料特性的MIC；通过电化学聚合法实现分子印迹过程，可以获得较为均匀、敏感的MIC膜材料。不同印迹策略的研究拓展了MIC的应用场景，丰富了MIC的种类，对新型、高性能MIC的开发具有一定指导意义。

## 2 分子印迹催化剂表征技术

为明确表征MIC空穴结构、成键特征以及催化剂理化性质，需要结合多种表征技术以解析和确认催化剂的结构。针对MIC的特征，我们简要介绍了几种典型的MIC表征技术。

电感耦合等离子体-原子发射光谱（ICP-AES） 在MIC的制备过程中，难以精确控制模板分子的洗脱程度，无法获得MIC内各元素的准确含量。目前，通常通过ICP-AES以精准测定MIC各类元素含量^［46，47］^。此外，得益于ICP-AES极低的检出限，ICP-AES还可用于辅助测定催化剂的稳定性^［17］^。然而，在利用ICP-AES测定催化剂元素含量时，会对MIC的结构造成不可逆破坏，且无法获得MIC所有的元素分布信息^［48，49］^。

元素分析（EA） 对于含有C、H、N等有机元素的MIC，可以通过EA分析测定其元素含量和比例，从而辅助MIC的结构解析^［25，41，50］^。然而，通过EA虽然可以得到MIC的详细元素含量，但是无法获得MIC中金属元素含量，无法获得各元素在MIC中的分布信息，且在表征过程中仍然可以对MIC造成不可逆的损伤。

热重分析技术（TGA） TGA常用于测定MIC的部分元素组成，可以实现样品组分高精度测定，获得部分有机物种及其含量等信息，是一种典型的MIC组分测定技术^［33，51］^。然而，利用TGA测定MIC组分时，测试时间较长，且无法获得MIC中元素分布信息，且对MIC样品造成不可逆的损坏。

核磁共振波谱法（NMR） NMR是一种基于原子核磁性质的非破坏分析技术，常用于定性分析MIC的元素成分和结构。目前，MIC的NMR表征中，通常捕捉分析^1^H、^11^B、^13^C、^29^Si和^31^P原子核的信号，以获得MIC中功能单体的化学环境以及MIC的微观结构^［31，46，52-54］^。

扫描电子显微镜（SEM） SEM是一种典型的MIC表面局部形貌表征技术。理想状况下，由于模板分子的洗脱，可以通过SEM观察到MIC表面均匀分布着粗糙的分子印迹空穴^［30，47，55-57］^。然而，通过SEM无法观察到金属MIC表面的活性金属纳米颗粒，无法获得MIC的表面细节参数。

透射电子显微镜（TEM） TEM常用于观察MIC表面局部形貌，在MIC表面局部区域形貌表征中，TEM可以弥补SEM的不足，有效表征MIC表面金属颗粒形貌，并提供金属颗粒粒径等相关信息^［30，33，47，57］^。因此，将SEM和TEM结合利用，可以实现对MIC局部表面形貌的有效表征。但是，因为SEM和TEM在表征过程中无法有效表征MIC整体形貌，因此需要结合其他表征技术，以获得完备的催化剂形貌参数^［58，59］^。

X射线衍射（XRD） 在MIC表征过程中，通过XRD可以获得催化剂的结构信息。XRD是一种高效、简便的MIC表征技术，以分子筛等晶体材料作为MIC载体时，通过XRD可以获得MIT对载体材料结构的影响，且可以定性获得分子印迹层的厚度等物理信息^［44，60］^。

傅里叶变换红外吸收光谱仪（FT-IR） FT-IR是一种典型的红外辐射吸收表征技术，常用于测定MIC中化学成分和结构信息。分子印迹空穴的形貌和成键模式对MIC的催化活性有直接影响。FT-IR可以高效、准确地表征出MIC内功能单体的成键模式，且可以通过成键模式一定程度上揭示MIC结构^［46，56，57，61］^。若条件允许，还可以利用原位红外表征技术，实现对MIC制备过程的有效监测和调控。

高分辨质谱（HRMS） HRMS拥有超高的分辨率和极低的检出限，可以精确测定样品的质荷比，常用于表征分析MIC微观结构和相对分子质量^［17，53］^。HRMS在表征过程中，主要包括离子化、质量分离和质荷比检测3个阶段。最终检测器捕捉不同离子信号，通过数据对比或理论计算即可确定MIC的微观分子结构。

X射线光电子能谱（XPS） XPS在对样品没有造成明显破坏的情况下即可提供分子结构和原子价态信息，且可以提供各种化合物的元素组成、含量、分子结构等信息。同时，XPS可以提供样品整体和局部微小区域信息，是一种广泛用于表征MIC元素和价态的先进表征技术。此外，通过XPS还可以获得金属MIC中金属成键配位形式，辅助调控MIC制备过程中的金属负载量，并指导MIC催化机理研究^［46，62］^。

其他MIC表征技术 除了上述几种典型表征技术外，紫外光谱（UV）、拉曼光谱（Raman）、N_2_等温吸脱附测试、X射线吸收精细结构谱（EXAFS）、理论计算等^［17，46，51，60，63-65］^催化领域重要的表征技术和理论计算分析手段也被用于MIC的结构表征，有效促进了高催化活性新型MIC的研发。

## 3 分子印迹技术在有机催化中的应用

分子印迹技术因其优异的分子识别能力而被广泛应用于催化材料的合成，通过MIT制备的催化剂可以有效提升催化反应效率，简化制备、分离、提纯等过程。在过去数十年中，MIC已经广泛用于诸多催化反应并展现出良好的催化性能和分子识别能力，尤其是在特异性识别和高选择性方面具有显著优势。为了更好地了解MIC的催化效果和应用，以下将按照反应类型（如水解反应、氧化反应、还原反应、偶联反应、聚合物反应器等）简要介绍MIC在催化反应中的应用。

### 3.1 水解反应

酯水解反应是最具代表性的水解反应之一（图3）。为了提高酯水解反应的效率，通常以目标产物的反应过渡态及其类似物为模板分子，以酰胺、咪唑、吡啶等含氮分子为功能单体制备高性能的MIC。过去20多年间，MIC在水解反应中的应用取得了良好的发展。邓云度等^［66］^通过MIT，以*N*-苯基-苯甲酰胺为功能单体，以甲基膦对硝基苯酯为模板分子，和ZnO共同构筑了富含三维孔道结构的MIC。实验和表征结果显示，该MIC在羧酸酯水解反应中展现出优异的催化活性，其内部的三维孔道结构显著增大了催化剂的比表面积，减少了底物进入分子印迹空穴的时间，提高了反应速率，获得了远超同期文献所报道的水解催化性能。但是该MIC催化活性对反应pH值较为敏感，在强酸性或碱性的反应环境中催化活性较低。Hradil等^［41］^以膦酸二苯酯为模板分子，*N，N*-二乙基（4-乙烯基苯基）脒为功能单体，采用悬浮聚合策略制备了具有高比表面积的聚合物微球MIC（图4）。作为对照，他们还制备了非分子印迹微球。该MIC具有优异的催化性能，在碳酸二苯酯和氨基二苯酯水解反应中的反应速率是溶剂自水解过程的100倍以上。进一步的研究表明，微球MIC的酯水解催化活性明显优于非分子印迹催化剂（NIC），其反应速率差异最高可达20余倍。

**图3:**
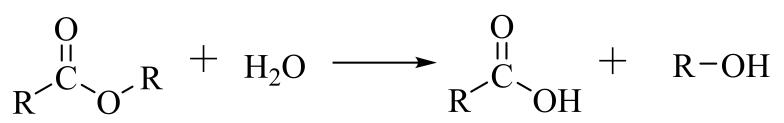
酯水解反应

**图4:**
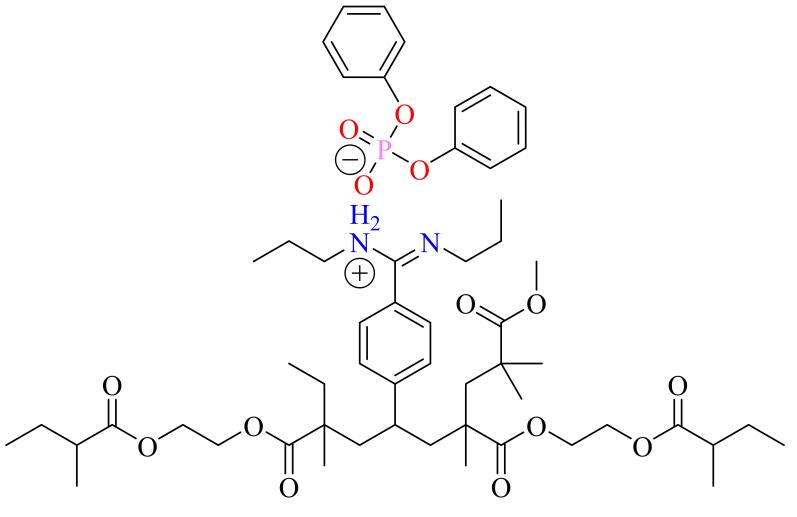
聚合物微球MIC的化学结构式

在MIC的制备过程中，模板分子和功能单体形成的印迹复合物结构越稳定，分子印迹空穴空间结构越明确，所得MIC分子识别性能越优异，催化反应的选择性越高。当使用过渡金属离子为枢轴进行自组装时，功能单体通过配位键与印迹分子相连接，显著增强了功能单体和印迹分子之间的相互作用，获得了结构更加稳定的印迹复合物，提高了MIC的分子识别能力。如图5所示，Li等^［55］^以配位能力较强的Co离子为枢轴，将1-乙烯基咪唑（VIm）功能单体配位在对硝基苯磷酸模板周围，构筑模板-枢轴-单体的强分子组装系统，制备了可特异性催化对硝基苯乙酸酯（NPA）水解的MIC-P。催化性能测试和动力学伪一级反应结果表明，醋酸正丁酯（BA）和NPA的水解率均随水解时间增加而增大，BA和NPA的自水解率最高为70%左右；与之相比，非分子印迹聚合物对BA和NPA几乎没有催化水解活性。模板-枢轴-单体组装策略可在一定程度上提高MIC的催化性能，是一种较有效的高性能MIC制 备策略。例如，以Co（Ⅱ）为枢轴制备的分子印迹聚合物MIC-P和常规方法制备的MIC均可特异性催化NPA水解，且MIC-P 水解活性稍高于MIC。此外，Luo等^［67］^以磷酸对硝基苯基钴二钠盐为模板，DVB为交联剂，VIm为功能单体，制备了具有酯水解反应催化活性的MIC。研究发现，以Co络合物为印迹分子时，制备的MIC刚性孔道结构更加稳定，可以更好地稳定目标产物过渡态，加速目标产物的生成，从而表现出更高的催化活性。应用分子印迹技术制备的催化剂具有极高的反应择形性，甚至可以催化目标手性产物的合成。

**图5:**
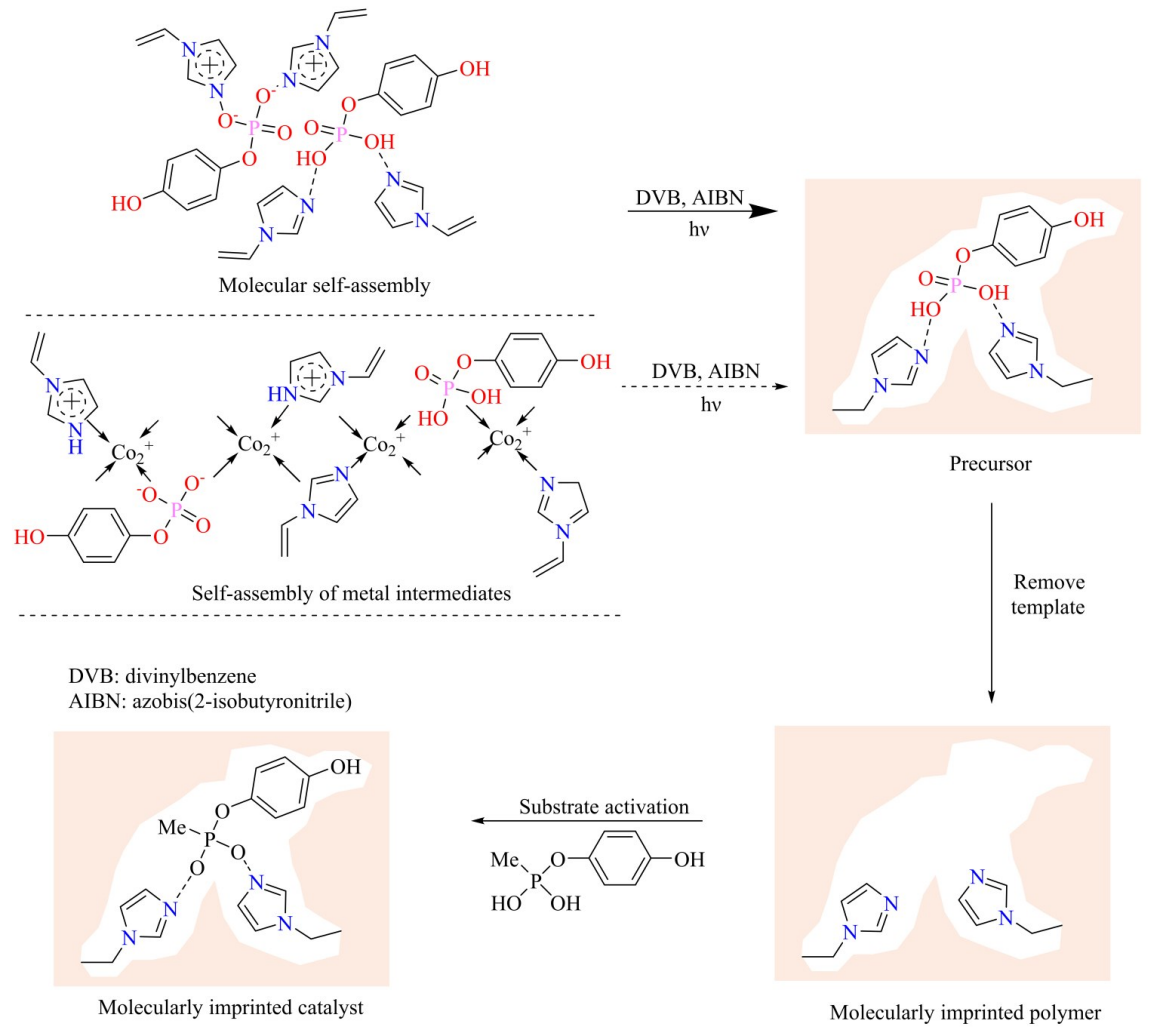
分子印迹催化剂的制备和分子识别示意图

Ohkubo等^［68］^通过自由基聚合法，以氨基酸酯水解反应过渡态类似物为模板，合成了MIC。他们制备的MIC在氨基酸酯催化水解反应中具有极高的反应选择性和产物择形性，所得水解产物具有优异的立体选择性，左旋产物（L型）和右旋产物（D型）之比最高为8.4。Guo等^［69］^采用“反应物-产物-双模板印迹”策略，制备了一种可以有效催化对羟基苯甲酸水解并消除水解产物的胶囊催化剂。在胶囊催化剂制备过程中，以4-硝基苯酚（4-NP）和对氧磷为模板分子，在乙烯基官能化SiO_2_表面组装后，与DVB交联聚合形成印迹聚合物，去除SiO_2_后得到目标MIC。该MIC的胶囊结构赋予了其优异的对羟基苯甲酸水解活性，水解反应的初始反应速率（*k*）为59.6×10^-2^ μmol/min，是SiO_2_除去前的近2倍（31.7×10^-2^ μmol/min）。此外，该MIC具有优异的稳定性，重复使用3次后催化活性无明显下降。Zhu等^［51］^采用分子印迹策略，制备了一种具有双分子印迹空穴的多孔芳香骨架MIC。该MIC以Zn（Ⅱ）复合物为活性中心，以对氧磷水解反应过渡态类似物1-（二乙氧基磷酰甲基）-4-硝基苯为反应的模板分子，以二甲基丙烯酸锌络合物和产物类似物4-NP的混合分子为产物吸附模板分子，以VIm为功能单体，在多孔芳香框架中与MAA交联聚合后制备了目标催化剂MICAFs。分子印迹空穴的存在赋予了MICAFs较好的底物选择性以及高于天然有机磷水解酶活性的对氧磷水解活性，为水解反应催化剂的构筑提供了新的思路。

### 3.2 氧化反应

氧化反应是一种常见的催化反应，近年来也发展了诸多性能优异的催化材料，其中在特定结构聚合物合成和选择性氧化方面，MIC表现出了较大的应用潜力。按照催化材料活性中心的金属特性分类，这类MIC可大致分为贵金属MIC、非贵金属MIC和无金属MIC 3类，以下将对其逐一介绍。

#### 3.2.1 贵金属MIC

钌离子络合物（如：［Ru（2，6-Cl_2_tpp）Cl_2_］、Ru（OH） *_x_* 等）是一类常见的氧化反应催化剂^［70，71］^，对有机氧化反应具有高催化活性。通过MIT制备的Ru基催化剂（Ru-MIC）具有优异的分子识别催化能力，具有优异的选择性和催化活性。图6展示了一种Ru-MIC的反应底物类似物制备策略。Severin等^［54］^以一种Ru的卟啉化合物1为催化活性中心，将1与氨基二苯甲烷络合制得模板分子2，在氯仿为致孔剂的条件下，采用反应底物类似物策略，将2与EGDMA聚合后脱除氨基二苯甲烷，得到了一种Ru-MIC。该MIC具有优异的催化氧化活性，可在不含无机酸的条件下催化氧化醇类化合物制酮类化合物。相较于非分子印迹Ru催化剂（Ru-NIC），MIT显著提高了聚合物的催化氧化活性，Ru-MIC催化二苯基甲烷氧化初始速率是Ru-NIC的6.4倍。同时Ru-MIC还具有优异的常温催化活性，在20 ℃时Ru-MIC催化二苯基甲烷氧化的初始速率是Ru-NIP的16.4倍。然而，由于在Ru-MIC制备过程中底物类似物仅通过单一配位键与Ru相连，分子印迹过程中底物类似物构型的结构不确定性较大，从而使制备的MIC分子印迹空穴结构的特异性降低，不利于获得具有优异分子识别能力和高催化活性的MIC。因此，为获得综合性能优异的MIC，可以通过多点结合策略制备催化剂。此外，在金属MIC的制备过程中，可以利用溶剂效应调节所得MIC的催化性能和选择性。当反应物极性差距较大时，可以利用溶剂（如含氟溶剂等）的诱导分配效应调控所得MIC的催化性能，这一现象与反应物自身性质无关^［64］^。Ru-MIC在生物有机催化氧化反应中依然具有较高的催化活性和反应选择性。Mizuki等^［62］^通过MIT合成了一种首次用于天然类固醇催化反应的Ru基卟啉化合物，该MIC可以在不保护3位羟基的条件下催化胆固醇-5-烯-3-醇及其衍生物（图7）的C5=C6发生环氧化反应，环氧化物的产率高达95%。

**图6:**
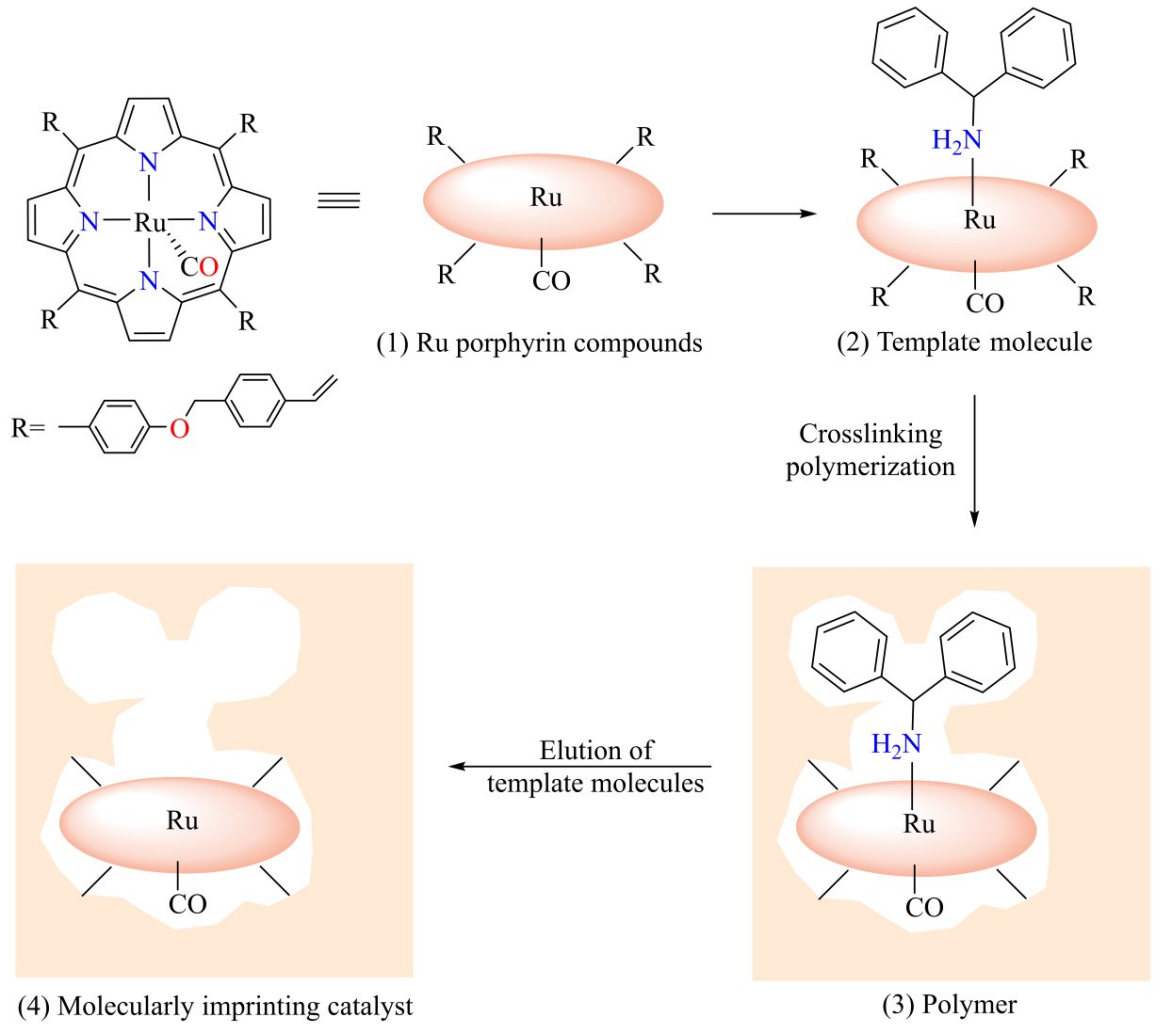
Ru-MIC制备示意图

**图7:**
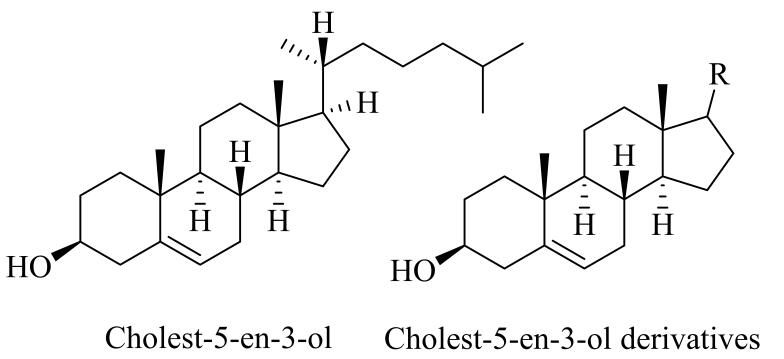
胆固醇-5-烯-3-醇及其衍生物结构式

苯甲醇选择性氧化反应是有机化工中的重要反应之一^［72-74］^，Au作为催化剂中的活性贵金属元素，Au基MIC对苯甲醇的选择性氧化反应展现出较高的反应选择性和催化活性。银董红等^［47］^以4-硝基苯甲醇与氯金酸形成的络合离子为模板，制备的Au/MIC具有一定的分子识别能力，可以在较温和条件下催化4-硝基苯甲醇氧化反应，获得75.6%的4-硝基苯甲醇转化率；而以非分子印迹金属聚合物Au/NIC为催化剂时，4-硝基苯甲醇转化率仅为41.5%。因此，经MIT处理后，Au/MIC催化活性显著高于Au/NIC。此外，Ni-MIC也是一种有效选择性催化苯甲醇氧化的贵金属MIC。Yuan等^［57］^以4-硝基苯甲醇和六水合硝酸镍的络合物为模板，丙烯酰胺为功能单体，与二甲基丙烯酸乙二醇交联耦合，制备了一种Ni基MIC。所制备的Ni-MIC展示出良好的4-硝基苯甲醇识别能力，在4-硝基苯甲醇的氧化反应中具有极高的选择性和催化活性。在实际氧化反应中，反应开始后1 h即可达到反应速率最大值，4-硝基苯甲醇的转化率高达72%，是非分子印迹Ni催化剂的1倍以上。

#### 3.2.2 非贵金属MIC

由于地球上丰富的储量，Fe基催化剂具有远超其他金属基催化剂的经济优势^［75-77］^，Fe基MIC也因此成为一种批量化、高性能MIC的催化材料。

如图8所示，Yin等^［78］^通过目标底物分子印迹策略，分别以邻、间、对硝基苯甲醇与FeCl_3_的配合物为模板，丙烯酰胺为功能单体，制备出对硝基苯甲醇具有高催化氧化活性的Fe（Ⅲ）-MIC，分别记作o-Fe（Ⅲ）-MIC、m-Fe（Ⅲ）-MIC和p-Fe（Ⅲ）-MIC。Fe（Ⅲ）的存在是该催化剂高催化氧化活性的原因之一，当30%的H_2_O_2_存在时，聚合物基质NIC对硝基苯甲醇几乎没有催化氧化活性，而Fe（Ⅲ）-NIC可以催化30%~50%的硝基苯甲醇氧化。MIT赋予了该Fe基催化剂更加优异的催化氧化活性和分子识别能力，经MIT处理后，在化学反应过程中，仅与Fe（Ⅲ）-MIP空穴结构匹配的底物可高效参与化学反应，催化反应的底物转化率高达76%，催化转化率最高为非分子印迹Fe基催化剂的2.5倍。

**图8:**
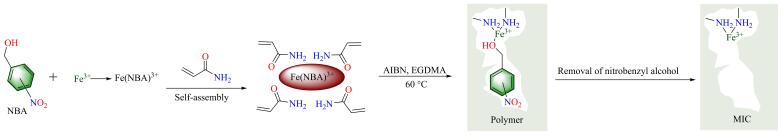
Fe（Ⅲ）-MIC制备示意图

袁新华等^［79］^采用底物分子印迹策略，以4-NP和六水合氯化铁络合物为模板，MAA为功能单体，二甲基丙烯酸乙二醇酯为交联剂合成了MIC的前驱体，在印迹分子的模板脱除和还原处理后，制得了对4-NP具有特异性识别能力的纳米铁基分子印迹聚合物（Fe-MIC），催化剂的制备流程如图9所示。该Fe-MIC具有大量与4-NP匹配的分子印迹空穴，可特异性识别、吸附和催化降解4-NP。催化性能测试结果表明，当pH值为3~7时，反应溶液的pH值对该Fe-MIC催化性能具有显著影响：随着pH值降低，催化剂催化活性增大，当pH值为3时，最高可有效降解80%的4-NP。分子印迹空穴的存在可以大幅提高该铁基催化剂对类Fenton反应的催化活性，Fe-MIC对4-NP的催化转化率比含铁的非分子印迹催化剂高出35%以上。

**图9:**
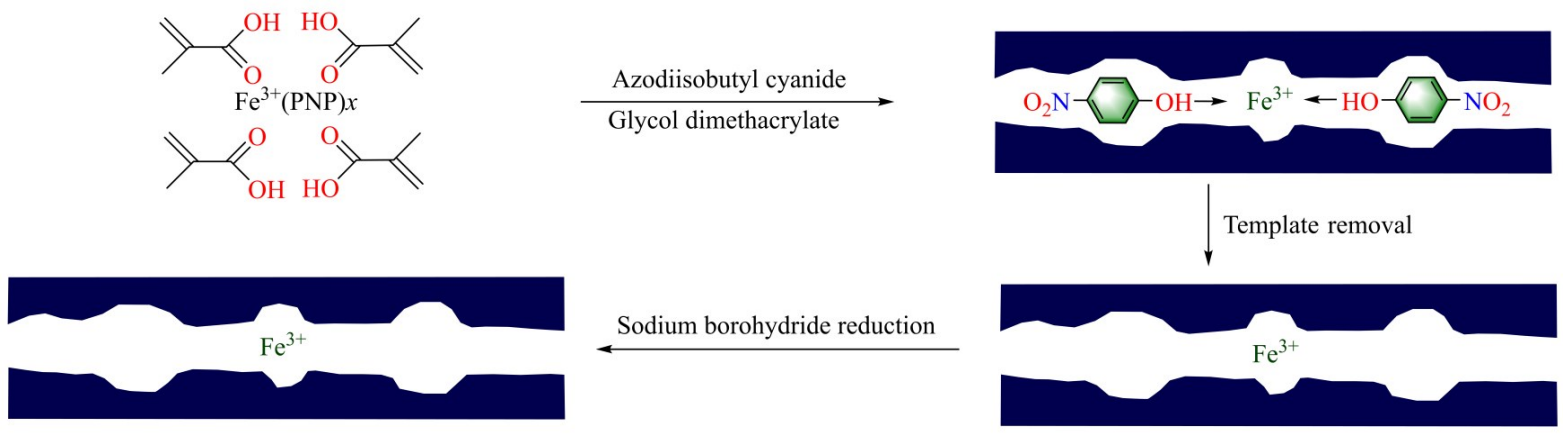
Fe-MIC制备示意图

SiO_2_作为一种稳定、常见的催化剂载体，通过MIT对SiO_2_的表面功能化处理后，可以制备出具有优异催化活性、特异性和稳定性的MIC。Tian等^［80］^通过表面分子印迹技术，对反应目标底物进行分子印迹，在氨基修饰的Fe_3_O_4_/SiO_2_核壳颗粒表面锚定对硝基苯酚分子印迹聚合物，制得了具有核壳结构的磁性分子印迹催化剂Fe_3_O_4_/SiO_2_-MIC。表面印迹处理增强了Fe_3_O_4_/SiO_2_-MIC与对硝基苯酚之间的结合作用，使催化剂表面具有更强的对硝基苯酚吸附能力。此外，分子印迹空穴的存在显著提高了催化剂的性能和反应的抗干扰能力。例如，Fe_3_O_4_/SiO_2_-MIC的对硝基苯酚表观降解速率常数为非分子印迹铁基催化剂Fe_3_O_4_/SiO_2_-NIC的2.8倍；在实际污水竞争催化降解系统中，Fe_3_O_4_/SiO_2_-MIC可有效降解75%的对硝基苯酚，而Fe_3_O_4_/SiO_2_-NIC受其他有机物的干扰，对硝基苯酚的降解率仅为40%。在利用类Fenton反应氧化有机化学物质降解的过程中，H_2_O_2_是关键氧化物，SiO_2_纳米颗粒表面柔软且具有特定的Si-O-Si键，易与H_2_O_2_形成氢键，是一种理想的功能单体。Chen等^［61］^采用目标底物分子印迹策略，以印染行业常用染料酸性橙Ⅱ（AOⅡ）为模板，通过MIT修饰Al^3+^功能化的SiO_2_，合成MIP纳米粒子，在其表面负载Fe^3+^离子后，制备得Fe/Al/SiO_2_复合物分子印迹催化剂（IMIPF）。研究表明，IMIPF对AOⅡ具有优异的分子识别能力、优先吸附能力和催化降解能力，AOⅡ降解效率高达92.85%。

此外，高比表面积的材料具有大量催化活性位点，是理想的催化剂载体。分子筛、金属有机框架材料（MOF）等多孔材料具有高比表面积，含有大量催化活性位点，可以在分子印迹过程中为印迹复合物提供更多锚定位点。且，由于它们稳定的化学性质^［81，82］^，以其为载体也可以提高MIC的稳定性。此外，在催化过程中，选用多孔材料作为载体可以筛分尺寸大于孔道直径的干扰分子，提高催化效率。因此，选用分子筛、MOF等高比表面积材料作为载体制备MIC是一种可行的策略。Yu等^［44］^以4A分子筛为载体，以亚甲基蓝为模板进行分子印迹，掺杂铁离子后制备了类Fenton反应的催化剂MI-FZ。分子印迹处理后的分子筛表面不再光滑，出现孔状结构，使分子筛的特异性吸附能力大幅提高。吸附性能测试结果表明，MI-FZ对亚甲基蓝的平衡吸附量约为Fe分子筛的3倍，且在混合溶液中也可以有效识别、吸附亚甲基蓝。催化性能测试结果表明，MI-FZ可以在室温下催化亚甲基蓝以一级反应进行类Fenton反应氧化降解，亚甲基蓝降解率高达92.2%。该MI-FZ具有优异的稳定性，在6次重复催化实验中亚甲基蓝降解效率仍可保持近90%。

核壳结构材料具有更高的比表面积，可以提高催化剂中目标底物的浓度，大幅提高MIC的催化效率^［83，84］^。Wan等^［37］^利用氢键、静电引力和*π*-*π*堆积疏水作用，以邻苯二甲酸二乙酯为模板，丙烯酸为功能单体，DVB为交联剂合成了MIP核心；随后在MIP表面生成铁基MOF材料MIL100，制得催化邻苯二甲酸二乙酯降解的MIL100-MIP催化剂。MIL100-MIP实现了邻苯二甲酸二乙酯的精确吸附，对邻苯二甲酸二乙酯的降解效率高达105.2 mg/g，在废水处理方面展现出巨大的应用潜力。除此之外，Wan等^［65］^也采用表面印迹策略，以磺胺甲噁唑为模板分子，加入*β*-环糊精预排列，再加入MAA功能单体和EGDMA交联剂，对Fe基MOF进行分子印迹修饰，制得可以有效识别、催化降解磺胺甲噁唑的核壳3D-MIC。得益于MOF对降解分子尺寸的筛分作用和分子印迹空穴对底物分子的识别作用，3D-MIC对磺胺甲噁唑的最大吸附量和降解率达到了276.71 mg/g和97%。该印迹策略赋予了3D-MIC优异的分子识别能力，在模拟天然废水、自来水、污染河水等复杂催化降解体系中仍可以保持较高的磺胺甲噁唑降解率，磺胺甲噁唑的降解率高达91%。

石墨烯等纳米材料具有稳定的理化性质和高比表面积，是一种高活性、高稳定性的MIC载体^［85，86］^。为了实现农药和化学品中有机磷神经毒剂（Ops，一种典型环境污染物）的有效氧化降解，Guo等^［87］^以UiO-66为载体，以轻质的石墨烯气凝胶为骨架，借助MIT，构筑了高活性、高稳定性的分子印迹催化体系。在分子印迹过程中，模板分子对氧磷与功能单体吡啶脒肟（PAAO）在Zn离子周围形成络合分子Zn-PAAO-对氧磷，随后形成具有特异性识别作用的分子印迹空穴，最终获得三维催化剂MOFs/MIC@GAs。三维多孔通道的存在为该MIC提供了优异的传质能力，分子印迹空穴赋予其优异的催化二甲基-4-硝基苯磷酸酯水解活性，反应动力学*k*_obs_和半衰期*t*_1/2_分别为0.222 7 min^-1^和3.11 min。此外，UiO-66框架赋予了MOFs/MIC@GAs优异的热稳定性和化学稳定性，该MIC最低碳化温度为420 ℃，在重复利用10次后，仍可保持93.5%的催化效率。

为进一步提高MIC的催化活性，在催化体系构筑过程中引入两种及以上活性金属是一种可行策略。Zhao和Ma^［60］^以Fe-MOF为基底，在掺杂金属后采用分子印迹策略，以目标降解物磺胺甲噁唑为模板分子，与功能单体丙烯酸组装后通过MIT，制备了一种三金属催化剂C-Fe-Cu-Mn-Nx@MIC。该MIC催化磺胺甲噁唑高效降解，且具有优异的稳定性，50 min即可催化95.87%的磺胺甲噁唑降解，重复使用7次后仍可催化80.67%的磺胺甲噁唑有效降解，为MIT在环境污染物催化氧化降解中的应用提供了新的思路。

#### 3.2.3 无金属MIC

在无金属存在时，MIC因其固定空间取向的空穴和特异性分子印迹催化活性位点，获得了优异的分子识别性能和催化氧化活性。Rychnovsky等^［88］^以2，2，6，6-四甲基哌啶衍生物为模板制备聚合物，然后选择性除去N取代基，获得具有N-O•催化剂活性位点空穴的MIC。该MIC对苄醇和脂肪醇均展现出优异的催化活性，且具有较高的稳定性，重复使用3次后催化活性无明显下降。

### 3.3 还原反应

在有机化学反应中，还原反应占据了重要地位。加氢还原反应作为有机化学中的代表性反应，被广泛应用于精细化工产品的制备和生物制药过程^［89，90］^，是众多学者研究的重点。因此，本文重点关注加氢还原反应，暂时不考虑CO、甲醛等还原性分子相关的还原反应。

#### 3.3.1 手性还原反应

在有机合成过程中，涉及对映异构体的产品合成和分离过程充满困难，合成的最终产物通常为对映异构体混合物，难以高效分离目标产物。MIC是一种理想的不对称合成反应催化剂，其表面富含具有特定空间取向的刚性空穴和特异性结合位点，这使其展现出优异的分子识别能力和催化性能，可以诱导反应进行，从而获得具有特定空间构型的产品。

Wulff等^［53］^以苯乙酮加氢还原反应过渡态类似物为模板，制备出一种含恶唑硼烷的MIC。该聚合物中富含的分子印迹空穴和特异性B-N键可以稳定反应过程中生成的扭船式过渡态，在硼烷-二甲基硫化物加成物还原苯乙酮的过程中有效催化合成目标产物（对映体超量（e.e.）值为82%）。

在过去数年间，含Ru、Rh等金属的MIC被广泛合成，以期获得具有优异对映选择性和催化活性的MIC。相较于均相催化剂，MIT构筑的非均相催化系统内含大量构型固定的分子印迹空穴，这赋予了该体系出色的分子识别能力，最终大幅提高了反应的选择性。其中，通过MIT，以均相催化剂为催化活性位点构筑催化体系，可以最大程度上保留均相催化剂的分子识别催化活性，是一种高性能MIC的有效制备策略。

Lemaire等^［11］^为避免在高压条件下使用易燃易爆的H_2_为还原剂，采用氢化物转移还原法还原苯基烷基酮，苯基烷基酮的氢化物转移还原机理如图10所示。为提高目标产物的收率，降低副产物的产量，该研究利用MIT，以催化前驱体［Rh（C_8_H_12_）Cl］_2_为Rh源，合成了Rh基MIC（Rh-MIC）。该Rh-MIC具有优异的催化活性和择形性，且催化活性远高于均相Rh催化剂。使用Rh均相催化剂催化苯乙酮氢化物转移还原反应时，7天后底物转化率达到100%，e.e.值为67%；以Rh-MIC为催化剂催化该还原反应时，在2天内便可定量还原苯乙酮，e.e.值高达70%。

**图10:**
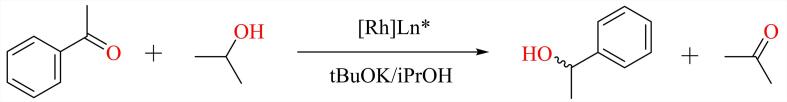
苯基烷基酮的氢化物转移还原

Polborn和Severin^［52］^同样证明以均相催化剂为MIC催化活性中心构筑催化体系是一种高效的MIC制备策略。如图11所示，他们以芳香酮加氢还原反应的均相催化剂CpRh为催化活性中心，通过膦配体与CpRh形成了一种具有分子内氢键的苯乙酮不对称氢化反应过渡态类似物，以该过渡态类似物为模板分子进行分子印迹，与EGDMA交联聚合，用氯取代膦配体后获得Rh-MIC。催化性能测试结果表明，Rh-MIC中由脱去膦配体形成的反应空腔赋予了该催化剂对苯乙酮的特异性识别能力。相较于其他与苯乙酮结构相似的酮底物，Rh-MIC可以精确识别苯乙酮，以苯乙酮为反应底物时该MIC具有最高的不对称催化氢化活性和对映选择性，目标产物转化率和e.e.值分别为81%和95%，底物转化率高出非分子印迹Rh催化剂40%。

**图11:**
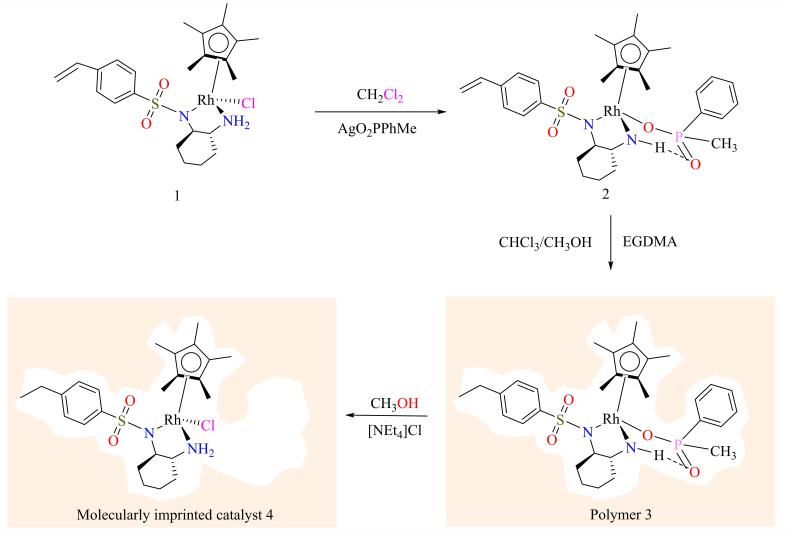
Rh-MIC不对称还原催化剂构筑示意图

不同的交联聚合方法制备的MIC内部分子空穴微结构和骨架稳定性存在较大差异，从而对MIC的催化活性造成影响。Tada等^［91］^为探究交联聚合方法对MIC催化性能的影响，在苯乙烯功能化SiO_2_表面负载Ru复合物，制备了一系列Ru-MIC，并对比了不同交联聚合工艺所得Ru-MIC的催化活性。不同的交联聚合策略决定了聚合物在功能化SiO_2_表面不同的堆叠方式，PH法和VDP法使聚合物均匀覆盖在SiO_2_表面，PP法则使聚合物无法完全覆盖SiO_2_表面。交联剂含量对不同聚合工艺获得的Ru-MIC的催化性能影响程度也不尽相同，相较于VDP-Ru-MIC，交联剂含量对PH-Ru-MIC催化性能的影响更为显著，表明VDP聚合过程中，聚合物单体首先与功能化SiO_2_表面苯乙烯分子发生反应，但进一步反应后聚合物脱离SiO_2_表面。此外，催化剂性能测试结果表明，PH聚合策略所得Ru-MIC具有最佳的催化活性，最高TOF可达99%以上，e.e.值最高可达91%，表明PH法制备的MIC为Ru复合物活性位点提供了足够的反应空间，对*o*-氟苯乙酮的不对称氢化反应具有优异的择形性和对映体选择性。

#### 3.3.2 非手性还原反应

加氢还原反应也被广泛应用于非手性产物合成中，是一种重要的有机产物合成策略。MIC因其优异的特异性催化能力，被广泛应用于催化非手性加氢还原反应。Iwasawa等^［46］^采用反应过渡态类似物印迹策略，通过化学气相沉积（CVD）技术，制备了含Rh的SiO_2_微孔分子印迹催化剂Rh_imp_。在Rh_imp_制备过程中，消除模板分子P（OCH_3_）_3_后在催化剂表面形成一个含有P（OCH_3_）_3_配体残基的分子印迹空穴。其中，空穴内Rh活性中心周围的P（OCH_3_）_3_配体残基通过表面氧原子以三齿形式和催化剂表面结合。该分子印迹空穴的存在大幅提高了催化剂的催化活性，相较于非分子印迹催化剂，Rh_imp_催化2-烯烃氢化反应活性提高了11倍。Tada等^［92］^以与邻氟二苯甲酮加氢还原反应的产物结构类似的Ru配合物为模板，在SiO_2_表面进行分子印迹，合成了一种含Ru的催化剂Ru-MIC-SiO_2_。Ru-MIC-SiO_2_具有优异的分子识别能力，可以精确识别酮底物种类和二苯甲酮衍生物转移加氢反应中F取代基位置和类型，且该MIC的分子印迹空穴壁上的NH_2_结合位点赋予了其优异的邻氟二苯甲酮不对称转移加氢还原产物吸附能力，提高了转移加氢还原反应活性，使该含Ru分子印迹催化剂具有优异的催化活性和反应择形性。Lin等^［93］^通过溶胶-凝胶法制备了具有不同含量的对苯二酚分子印迹空穴的介孔SiO_2_，并在该SiO_2_中负载一定量的Ag纳米颗粒，可以制备出对苯酚衍生物加氢还原反应具有高催化活性的Ag-MSN-MIC。该催化剂中，MSN丰富的介孔和高比表面积有利于Ag纳米颗粒的吸附和负载（最高Ag负载量为3.5%，平均粒径为2 nm）。催化性能测试结果表明，当以NaBH_4_为助催化剂时，可在5 min内催化4-NP完成加氢还原反应。催化剂元素含量分析和形貌表征结果显示，相较于非分子印迹催化剂，分子印迹空穴的存在显著减小了Ag纳米颗粒的粒径，使其暴露出更多的催化活性位点，且较小粒径的Ag纳米颗粒不会堵塞催化剂内的介孔，利于内部物质扩散。

目前，多采用交联聚合策略制备传统MIC，所得MIC为高分子催化剂。为进一步提高MIC的制备效率，彻底解决模板分子难以完全洗脱的难题，Ordomsky和Khodakov等^［16］^提出了一种表面纳米岛修饰策略，制备了具有高活性、高选择性的Pd-MIC。

如图12a所示，Pd-MIC以苯或苯的衍生物为模板分子，将模板分子吸附到对苯及其衍生物的加氢还原反应具有催化活性的Pd金属表面，随后使用二甲氨基丙胺（dimethylaminopropylamine，DMAPA）毒化多余的Pd催化活性位点，最后脱附模板分子，在Pd表面形成对特定分子具有催化加氢还原活性的纳米岛，制得Pd-MIC。该MIC在催化氢化过程中，具有特定分子结构的活性岛赋予了Pd-MIC良好的分子结构/尺寸选择能力。在反应过程中，只有形状和大小与活性岛相近的特定分子才可以被该MIC催化氢化，若底物分子体积过大，则无法有效吸附到活性岛，无法催化反应发生。催化剂性能测试结果表明，表面吸附印迹策略制备的MIC具有优异的底物选择性，以苯为模板分子制备的催化剂Ben-Pd-MIC对苯的转化率高达97.3%，苯的选择性超过98%。

**图12:**
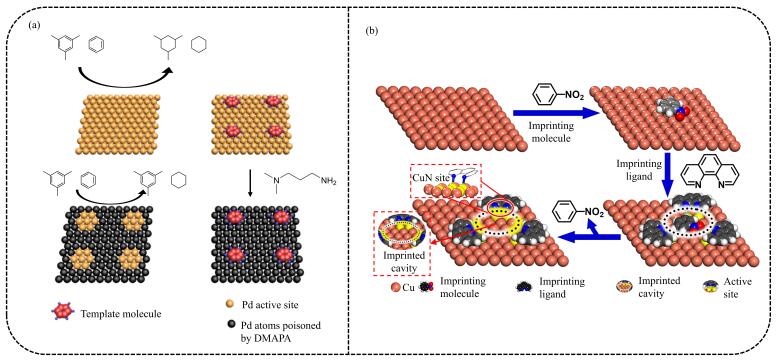
表面印迹催化还原催化剂示意图

虽然上述表面吸附印迹策略可以实现MIC的有效制备，但是该策略导致了大量催化活性位点的浪费，最终致使催化剂活性大幅降低。Shi和Cui等^［17］^同样证明了表面吸附印迹策略的可行性，并且实现了分子水平的催化活性位点构筑。如图12b所示，该体系通过在Cu/Al_2_O_3_表面上依次吸附印迹分子（N，如硝基苯及其衍生物）和印迹配体（L）的方法成功制备了一系列分子印迹催化剂（Cu/Al_2_O_3_-N-L）。该类催化材料对硝基化合物中的印迹分子表现出较高的加氢活性和独特的分子印迹效应。通过结构表征和密度泛函理论（density functional theory， DFT）计算发现，该催化体系具有优异分子印迹效果的原因在于形成了新的活性Cu-N位点和精确的分子印迹空穴。该分子印迹催化剂可以重复使用多次且相应的分子印迹功能得到保留。此外，通过更换印迹分子可以制备出多种用于其他硝基化合物高选择性加氢的分子印迹催化剂，表明该方法具有较好的普适性。这一体系表现出了活性位点结构明确、易于循环利用、制备方法具有一定普适性等优势，为合成具有新活性位点和精确印迹腔结构的MIC提供了一种新的思路。

### 3.4 偶联反应

偶联反应是一种常见的有机化学反应类型，是一类将两个化学单位有机分子反应生成一个化学单位有机分子的反应统称^［94，95］^。在有机化学过程中，C-C偶联反应和C-N偶联反应是两类被广泛应用的偶联反应。MIT作为一种有效的催化剂制备策略，其制备的MIC材料已广泛应用于C-C、C-N偶联反应中。

#### 3.4.1 C-C偶联反应

在传统有机化学过程中，通过C-C偶联反应可以实现碳链的可控增长或碳链重整，是一类基本的目标产物制备方式。自20世纪90年代末开始，分子印迹技术被广泛应用于C-C偶联反应，现已成功制备出大量对C-C偶联反应具有优异反应择形性和催化活性的MIC。

金属MIC 为获得对C-C偶联反应具有高催化选择性和特异性的MIC，在分子印迹过程中通常使用Co、Pd、Al等金属合成MIC。如图13所示，Mosbach等^［10］^通过MIT，在4-VPy-苯乙烯-二乙烯基苯共聚物中对二苯甲酰甲烷和Co（Ⅱ）离子复合物修饰改性，合成了一种可以选择性催化苯乙烯酮和苯甲醛缩合偶联的Co基MIC催化剂（DBM-Co^2+^ MIC），该MIC拥有远高于均相催化剂的催化活性和稳定性，持续运转数周后仍对缩合偶联反应保持较高的催化活性。为制备“人工活性位点”，Gagné等^［96］^以Pd为催化活性位点，首次将过渡金属配合物印迹到苯乙烯、DVB或二甲基丙烯酸乙酯的聚合物网络中。该Pd-MIC中构筑的Pd人工活性位点处具有手性空穴，且空穴的拓扑结构受金属单体上可以移动的模板分子构型影响，实现了空穴结构的精准合成。催化剂性能测试结果表明，该Pd-MIC催化丙烯基烷基化反应的转化率高达95%。银董红等^［56］^以2-乙酰基环己酮与Co（Ⅱ）的配合物为模板，合成了对苯甲醚乙酰化反应的邻位取代产物有一定催化活性的含Co催化剂Co（Ⅱ）-MIC。Murashkevich、Fedorova和Kuznetsova等^［45］^结合溶胶-凝胶法和分子印迹技术，合成了一种含芳香族羧酸分子印迹空穴的羧酸复合材料催化剂SiO_2_-TiO_2_-MIC。催化性能测试结果表明，该MIC对Biginelli反应具有优异的催化活性和产物立体选择性，相较于无催化剂时，目标产物的产率提高了20~30倍，e.e.值提高了2倍以上。

**图13:**
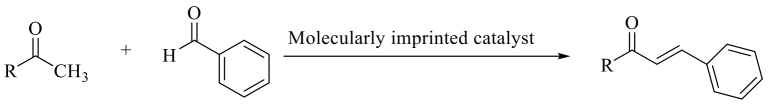
苯乙烯酮-苯甲醛C-C偶联缩合反应示意图

无金属MIC 不含金属的MIC在C-C偶联反应中常用于催化异构化和Diels-Alder环加成反应，促进碳链结构的改变，以获得多种类型的有机化工产品。苯并恶唑异构化反应如图14所示，Liu和Mosbach团队^［97］^对苯并恶唑异构化制邻苯酚氰反应过渡态类似物进行分子印迹，制备了一种具有Lewis碱催化位点的MIC。该MIC的分子印迹空穴和Lewis碱性结合位点使其对苯并恶唑异构化反应具有优异的底物识别能力和催化活性，MIC催化苯并恶唑异构化反应速率为未经分子印迹处理的聚合物基质的7.2倍，且表现出Michaelis-Menten动力学特征。相较于未催化的游离吡啶的二级速率常数，在MIC存在时反应速率提高了40 000倍。此外，Motherwell等^［98］^选用*α*-氧化蒎烯氧化异构化反应产物类似物的Lewis酸为印迹分子，与苯乙烯/DVB交联聚合，制得可以催化*α*-氧化蒎烯氧化异构化为*trans*-香芹酚的MIC。Visnjevski、Yilmaz和Brüggemann^［34］^采用无溶剂策略，将六氯环戊二烯与马来酸的Diels-Alder环加成反应过渡态类似物氯苯酸酐接枝到功能化SiO_2_表面，与功能单体MAA和交联剂DVB交联聚合后，溶解SiO_2_获得MIC壳层。该MIC壳层可以降低六氯环戊二烯与马来酸的Diels-Alder环加成反应的活化能，对该反应具有催化作用。此外，该团队还发现，在釜式反应器和固定床反应器中，通过该策略制备的MIC壳层具有优异的稳定性，可以在极端反应条件下催化生成目标产物^［99］^。另外，Nicholls等^［31］^以1，3-丁二烯氨基甲酸苄酯和*N，N*-二甲基丙烯酰胺反应的endo-和exo-产物的过渡态为模板分子，与丙烯酸和DVB交联反应，制得具有分子识别能力且对Diels-Alder反应具有优异催化活性的MIC。研究表明，相较于溶液反应，该MIC最高可使反应速率提高20倍。

**图14:**
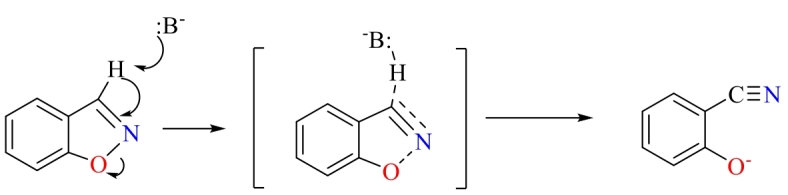
苯并恶唑异构化反应

#### 3.4.2 C-N偶联反应

芳香硝基化合物是一种重要的化工原料，现已被广泛应用于制备染料、香料等精细化工产品^［100-102］^。通过C-N偶联反应，可以实现芳香硝基化合物的简易、高效制备。尽管目前已开发多种苯及其衍生物的硝化反应路线与方法，但其分子内选择性仍未达理想水平，难以满足实际应用需求^［103，104］^。MIC具有优异的产物择形性，可以大幅提高C-N偶联反应过程中特定构型产物的高效制备。李雷和李明玲^［105］^以4-NP为模板，采用目标产物分子印迹策略，通过正硅酸乙酯水解反应，制备了分子印迹聚硅氧烷微球（MIPM）。在MIPM的制备过程中，4-NP用量可以显著影响MIPM的催化活性，若4-NP用量过高，硅氧烷微球易发生团聚，所得MIPM比表面积减小，催化活性降低。研究表明，通过该工艺制备的MIPM在较低温度下即可获得理想的催化活性和分子内选择性，最高底物转化率为81.4%，邻、对位产物比为1.46。

### 3.5 聚合物反应器

虽然金属纳米颗粒具有优异的催化活性，但是其较高的表面活化能使其容易团聚形成金属团簇，难以单独稳定存在^［106，107］^。将金属纳米颗粒封装在聚合物中制成聚合物反应器可以有效防止颗粒团聚，优化金属纳米颗粒性能。通过不同的聚合策略，选用不同的聚合单体，可以制得具有多种功能特性的聚合物反应器。为提高催化反应效率，降低化学反应成本，还可以在同一聚合物反应器中加工不同反应的催化活性位点，以获得对多种化学反应均有催化活性的级联反应器。一般而言，不同反应过程之间存在相互干扰的现象，将不同催化反应过程的催化位点简单地相互结合无法有效催化多步反应^［108，109］^。因此，在级联反应器的制备过程中，通常在反应器内部将不同催化过程分离，使不同催化过程高效、有序地进行。MIT可以赋予级联反应器优异的分子识别能力和特异性催化活性，使级联反应器精确、高效、有序地催化不同反应。此外，金属纳米颗粒的封装和MIT的处理使聚合物反应器具有较高的比表面积和孔体积，为级联反应提供更多的催化活性位点，促进了反应器内部的传质，促使反应可以连续、高效地有序进行，提高了最终产物的制备效率。

如图15所示，Yan和Wang等^［110］^将Ag纳米颗粒（AgNP）、级联反应过渡态类似物磷酸对硝基苯酯和4-NP、聚乙烯基咪唑自组装形成印迹复合物后，在二乙烯苯中交联聚合制备用于催化NPA水解-还原级联反应制4-氨基苯酚（AP）的分子印迹聚合物反应器MIC-NPA-AgNP。因为AgNP的存在，MIC-NPA-AgNP通常具有较大的立体尺寸，利于反应底物传递和产物脱出。得益于MIT，在级联反应过程中，不同反应物经分子印迹空穴调整进入位点，使得整个反应能够连续、高效进行。当NaBH_4_存在时，MIC-NPA-AgNP对级联反应NPA水解-还原制AP具有优异的催化活性，底物转化率为常见分子印迹催化剂MIC-AgNP、非分子印迹含银催化剂NIC-AgNP和非分子印迹催化剂NIC的两倍以上，证明了MIT与聚合物反应器构筑相结合策略的可行性。

**图15:**
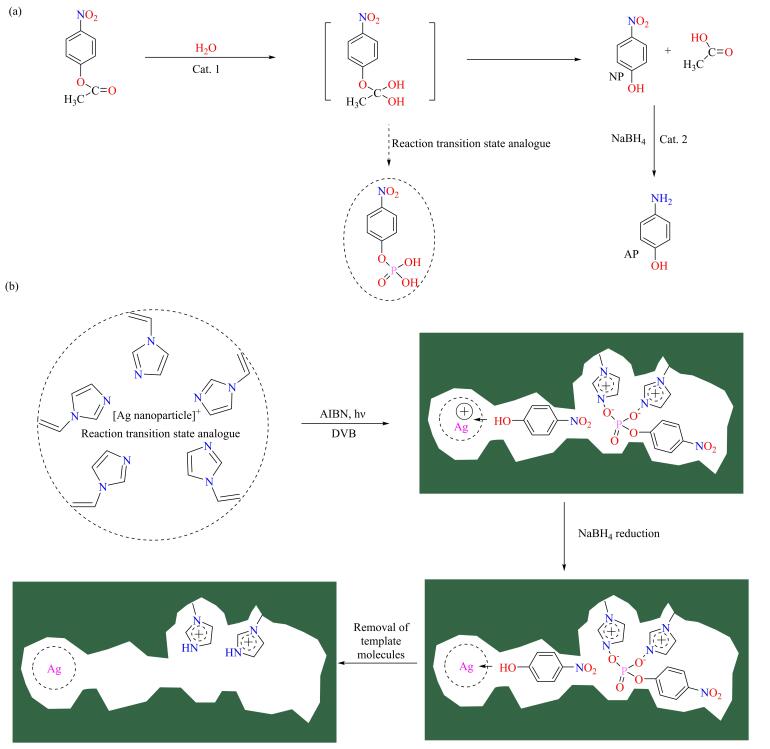
MIC-NPA-AgNP的（a）反应机理图和（b）分子印迹示意图

Li等^［29］^以含酸性催化位点的双功能MIC和铂纳米颗粒（PtNP）为原料，构筑了级联反应器MIC-NPA-PtNP。在该级联反应器中，MIT和PtNP的负载使MIC-NPA-PtNP具有更高的比表面积和孔体积。由于分子印迹酸性催化活性位点的存在，对4-硝基苯乙酸酯水解-还原制AP的级联反应催化活性显著高于非酸性MIC-NPA-PtNP和非分子印迹级联反应系统。且MIC-NPA-PtNP具有优异的稳定性，在一系列反应循环后催化活性不发生明显下降。为获得可有效连续催化反应的功能反应器，Li等^［30］^采用双模板分子印迹策略，分别以双（4-硝基苯基）碳酸酯（BNPC）以及4-NP为模板剂，以AMPS为功能单体，在MBA中交联聚合，制得对BNPC水解-还原级联反应具有优异的催化活性和稳定性的双层分子印迹级联反应器MIC-AuNP-BNPC。催化剂形貌表征结果表明，MIT赋予了催化剂更多的分子印迹空穴，增大了催化剂表面粗糙度，为反应提供了更多的催化活性位点。催化剂性能测试结果表明，分子印迹策略可以显著提高催化剂活性，两步分子印迹策略为级联反应构建了有效的物料传递通道，相较于非分子印迹Au纳米粒子聚合物，底物转化率提高了26%，底物转化率高达66%。

为进一步提高级联反应器催化级联反应的活性，以温敏材料为反应器载体，通过反应过程中的温度变化实现催化反应的智能切换是一种理想的反应器设计策略。为获得具有温度自响应能力的智能反应器，Li等^［111］^受贻贝等海洋生物的启发，设计了一种仿生智能反应器，如图16所示。在该反应器制备过程中，在AgNP的级联反应器中引入多巴胺分子，通过MIT制备了一种具有独特的分子印迹层和夹层开关的级联反应器MICAgPRS（其中：MIC代表分子印迹催化剂，Ag代表银纳米颗粒，PRS代表聚合物反应器开关）。MICAgPRS对NPA水解-还原制AP的级联反应具有优异的催化活性。该级联反应器的溶胀率随温度升高而增大，随着NPA的催化水解，反应温度逐渐升高，当温度高于38 ℃时，聚合多巴胺（PDPA）和聚丙烯酰胺（PAm）层PDPA-Pam解离，级联催化通道开放，由NPA水解生成的对硝基苯酚进入MIPAgPRS第二层发生催化还原反应，最终NPA转化为对氨基苯酚。Li等^［112］^参考了茅膏菜的“猎食”行为，发展了从“分子捕捉、初次分解”到“再消化吸收”的双功能层级联反应器SVA-MIC-Ni。该反应器第一层由具有类似拉链的活性VIm和AMPS共聚物组成，在不同反应温度下控制反应器活性位点裸露或隐藏。当反应温度较低时，反应器第一层关闭，无法捕获并催化水解4-硝基苯乙酸酯NPA；当温度较高时，第一层催化反应位点裸露，对NPA特异性识别，对级联反应NPA水解-还原为AP具有催化作用，NPA转化率约为75%。此外，Li等^［113］^通过MIT，设计了一种可在高温下“解冻”形成“流动域”的智能反应器。在反应器制备时，他们以Ag-NPA为模板，与2-壬烯酸、2-丙烯酰胺基-2-甲基丙磺酸、*N，N′*-亚甲基双丙烯酰胺交联聚合，制得了对温度敏感且具有长链换向特性的双层级联反应器SNA-MIC。温度较低时，表层分子印迹层对反应底物具有分子识别能力，在反应器内部催化NPA水解，生成的4-NP随即进入还原层，在还原剂存在时被AgNP催化还原为AP；当温度较高时，SNA-MIC第一分子印迹层“解冻”形成“流动域”，失去对反应底物的分子识别能力。根据不同底物的特点，Li等^［114］^以反应底物草酸双（2，4-二硝基苯基）草酯为模板分子进行分子印迹，制备了一种对草酸双（2，4-二硝基苯基）草酯水解-还原级联反应具有催化作用的含Ag纳米颗粒的温敏自响应反应器SVI-AmAg-MIC。当反应温度较低时，该反应器通道打开，草酸双（2，4-二硝基苯基）草酯在SVI-AmAg-MIC的酸性催化位点发生水解反应，生成2，4-二硝基苯酚，随后进入分子印迹层被还原为2，4-二氨基苯酚；当反应温度高于38 ℃时，反应器通道层关闭，草酸双（2，4-二硝基苯基）草酯直接进入分子印迹层发生还原反应，生成草酸双（2，4-二氨基苯基）草酯。

**图16:**
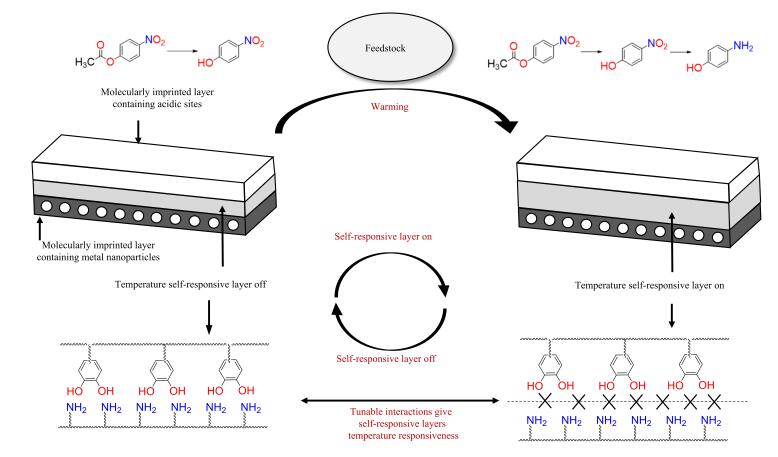
MICAgPRS级联反应器原理示意图

由此可见，在高性能高分子级联反应器的构筑过程中，利用MIT制备金属纳米颗粒负载的反应器可以避免级联反应中不同催化过程之间相互干扰，提高连续反应效率。在反应器构筑过程中，针对级联反应的不同催化过程分别进行分子印迹，可以为反应器提供更多的催化活性位点与物料传递通道，显著提高级联反应效率。此外，将MIT与先进功能高分子技术相结合，赋予反应器环境响应能力，拓展了高分子级联反应器应用场景，提高了反应器催化反应效率，增加了最终产物产率，是一种理想的功能高分子级联反应器制备策略。

## 4 分子印迹技术在光/电催化中的应用

光/电催化作为一种高效的催化技术，在污染物降解、温室气体转化及特殊结构化合物合成等领域具有显著优势^［115-118］^。基于MIT制备的光/电MIC，其表面高密度印迹空穴的存在显著提高了光/电催化剂的催化活性和抗干扰性，使其在复杂反应体系内仍可以对特定目标底物具有较高的催化活性。此外，催化剂表面具有特定空间构型的印迹空穴不仅提高了催化剂的比表面积，而且提高了其对特定反应底物的吸附能力，这也一定程度上提高了目标反应的反应效率。

MIT是一种有效的高活性、抗干扰光催化剂制备技术。García、Serre和Mouchaham等^［119］^以HCOOH为模板分子对Ti-MOF进行分子印迹，制备出了Ti基MOF分子印迹光催化剂（Ti-MOF-photo-MIC）。催化性能测试结果表明，Ti-MOF-photo-MIC具有优异的光催化甲酸分解制氢活性，Ti-MOF-photo-MIC光催化活性远高于MIL-125（Ti）和UiO-66（Zr），且表观量子产率高达22%。Liu等^［120］^采用MIT，通过水热合成法制备了一种具有优异化学稳定性的分子印迹光催化剂Fe_3_O_4_/g-C_3_N_4_（MI-FC）。分子印迹空穴的存在使MI-FC对环丙沙星和左氧氟沙星具有优异的吸附能力和光催化降解活性。MI-FC可以选择性吸附并光催化环丙沙星降解，对环丙沙星的吸附和光催化降解效率均高于左氧氟沙星（环丙沙星的吸附和光催化降解效率分别为51.18%和71.44%）。Fiorenza等^［18］^为除去自然水体中的特定农药，通过凝胶-溶胶法制得TiO_2_分子印迹光催化剂。该研究分别以常见农药除藻剂2，4-二硝基苯酚和杀虫剂吡虫啉为模板进行分子印迹，并测试所得光催化剂对上述两种农药的催化降解性能和选择性。结果表明，在紫外光照射条件下，所得TiO_2_分子印迹光催化剂对水体中污染物种类显示出优异的选择性，在光照180 min时对2，4-二硝基苯酚的降解效率超过45%，对吡虫啉的降解效率高达40%。Luo等^［121］^以甲基橙为模板分子，通过MIT向催化剂表面引入大量分子印迹空穴，成功制备出一种Ti基聚吡咯分子印迹光催化剂MIC-PPy/TiO_2_。分子印迹空穴的存在显著提高了该Ti基光催化剂的催化活性，MIC-PPy/TiO_2_的催化活性为非分子印迹Ti基催化剂的两倍，该Ti基分子印迹光催化剂制备流程如图17所示。

**图17:**
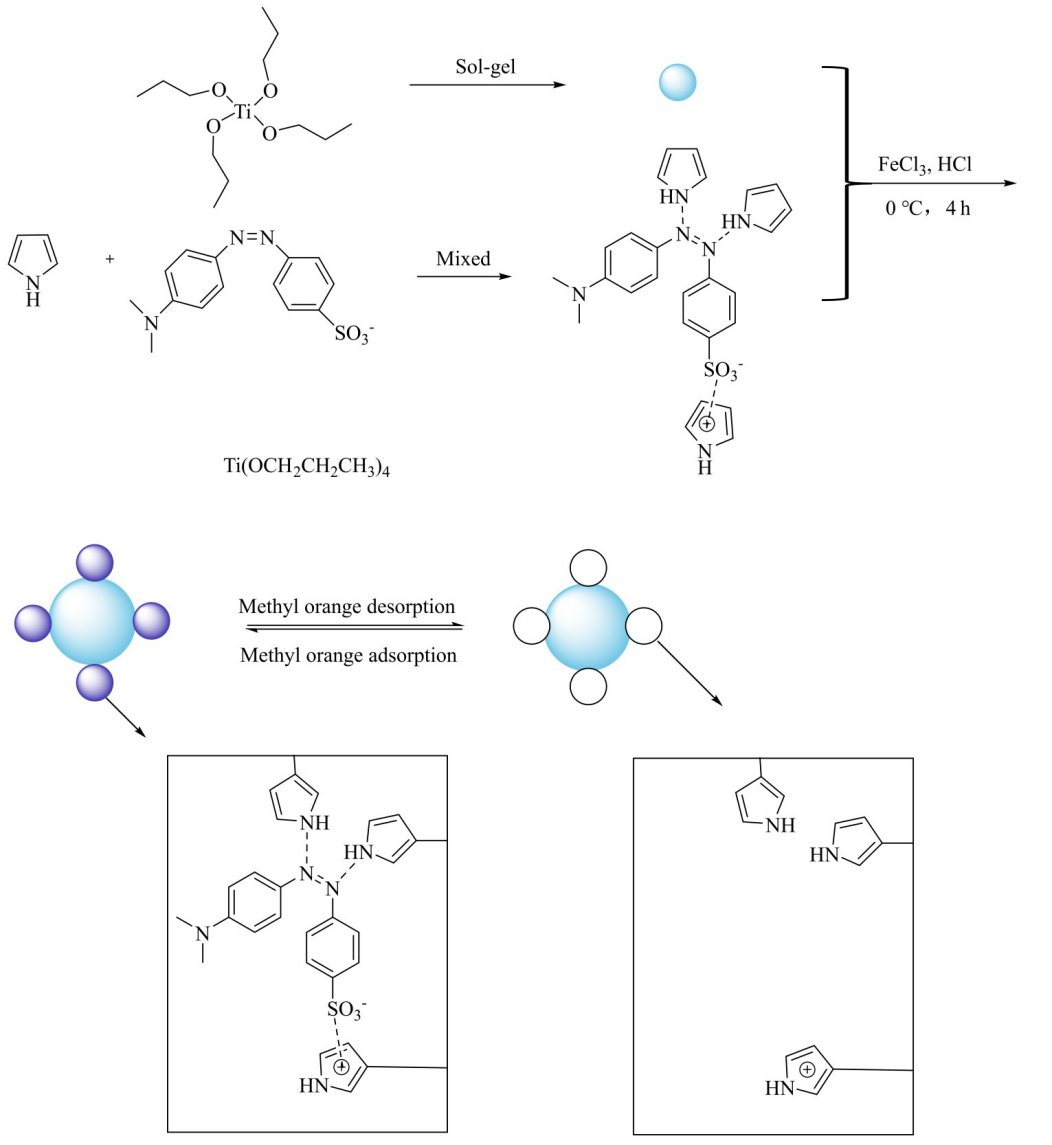
MIC-PPy/TiO_2_光催化剂制备流程图

如今，MIT已被广泛用于具有分子识别催化性能的电催化材料制备。MIT可以大幅提高电极的比表面积和选择性吸附性能，促使电极特异性吸附特定底物，从而提高特定反应的发生。通过MIT处理电催化剂，可以提高催化剂对特定底物的特异性吸附能力，增加催化剂表面底物浓度，促进目标产物的生成。Yan和Wang等^［15］^借助三苯基甲烷-4，4′，4′′-三异氰酸酯（TTI）与1*H*，1*H*，9*H*，9*H*-全氟-1，9-壬二醇（PFND）的亲核加成反应，以高浓度氮为模板进行分子印迹过程，制备了一种用于电催化合成氨工艺的全氟分子印迹材料PFMI。该分子印迹材料具有优异的氮识别能力，即有机骨架中强极性的C-F键使骨架中电荷分布不均，引发氮定向转移和富集。在电催化合成氨过程中，以PFND为电催化剂的吸附层可以有效富集氮元素，促进电化学氮还原反应的发生。催化剂性能测试结果表明，相较于裸露的催化剂，PFND的包覆使电催化剂的氨生产率（185.7 μg/（h·mg））和法拉第效率（72.9%）均提高了约3倍。

Zhao等^［122］^采用双分子印迹策略，制备了一种（001）晶面的二维光电催化剂（DMIPEC）。在DMIPEC制备过程中，Zhao等^［122］^分别使用2，4-二氯苯氧乙酸（2，4-D）和2-（2，4-二氯苯氧）丙酸（2，4-DP）为模板进行分子印迹。双分子印迹空穴的存在显著提高了2，4-D和2，4-DP的吸附效率，大幅提高了电极表面目标降解物的浓度。此外，得益于双分子印迹空穴对目标污染物的选择性吸附富集作用以及其对目标污染物的选择性催化降解作用，DMIPEC对目标污染物具有优异的选择性降解活性。催化性能测试结果表明，在复杂污染体系中，DMIPEC对2，4-D和2，4-DP具有优异的选择性催化降解活性，2，4-D和2，4-DP的去除率高达90%。此外，Zhao等^［123］^以2，4-D为模板分子，通过MIT，制备出了一种分子印迹介孔SnO_2_电催化电极（MIE-SnO_2_，图18）。SnO_2_电催化电极表征结果表明，相较于传统SnO_2_电极，MIE-SnO_2_的介孔结构赋予了其更高的BET表面积和电化学有效表面积，同时也赋予了MIE-SnO_2_更高的-OH生成能力，从而提高了MIE-SnO_2_的电化学活性。催化性能测试结果表明，MIE-SnO_2_可有效催化2，4-D的降解，6 h即可使污水中的2，4-D含量降至54 ppb。此外，虽然印迹空穴的存在不影响MIE-SnO_2_的电化学活性，但是印迹空穴赋予了MIE-SnO_2_优异的分子识别性能，可以使2，4-D在MIE-SnO_2_表面优先吸附和富集。

**图18:**
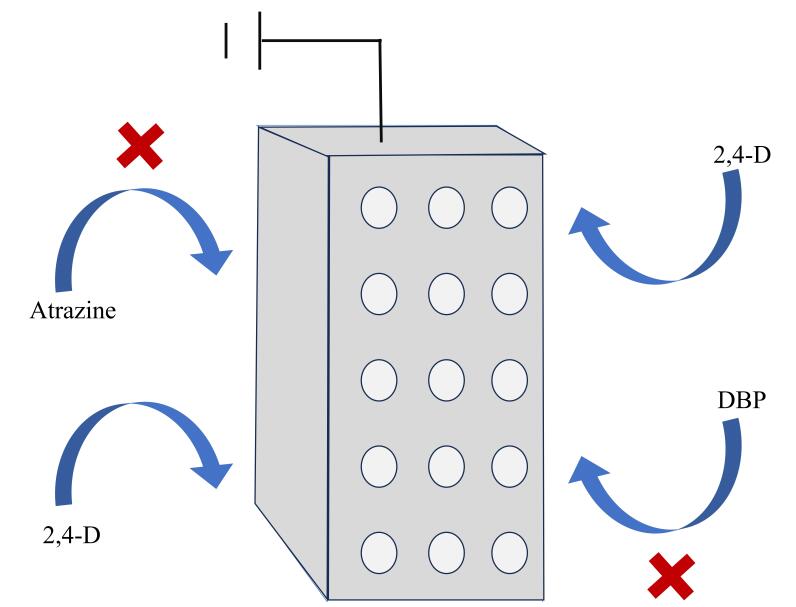
MIE-SnO_2_电催化电极选择性识别吸附示意图

## 5 分子印迹技术在酶催化中的应用

生物酶的高活性和专一性掀起了人工酶合成的热潮^［124-126］^，由MIT制备的人工酶催化剂具有特定的反应位点和分子印迹空穴，可以大幅提高催化剂的分子识别能力和选择性^［127］^。

Bose和Zhao等^［38］^报道了一种表面-核心双交联胶束分子印迹改性方法，开发出一种适用于人工酶的MIC制备策略。表面-核心双胶联胶束分子印迹策略是一种适用于多种类型人工酶合成的普适性方法。通过该策略制备的人工酶实现了对烯烃的原子级识别，可以选择性催化烯烃环氧化反应。Erdoğan Özgür^［128］^以CO_2_为模板分子，利用可聚合的L-组氨酸衍生物（MAH）及Zn^2+^的L-组氨酸的复合物制备了一种催化CO_2_水合反应的人工酶。催化性能测试结果表明，该人工酶可以有效催化CO_2_的水合反应，且随着Zn^2+^含量的增加，该人工酶对CO_2_水合反应的催化活性增强，反应速率明显提高。Devaky等^［129］^通过MIT，以苯丙氨酸对硝基苯胺的酰胺解反应类似物1-（*N*-苄氧羰基氨基）-2-（苯基）乙基膦酸苯酯为模板分子，以*N*-甲基丙烯酰-L-组氨酸、*N*-甲基丙烯酰-L-天冬氨酸和*N*-甲基丙烯酰-L-丝氨酸为功能单体，在EGDMA中交联聚合，合成了一种糜蛋白酶模拟物MIAE（molecularly imprinted artificial enzyme）。人工酶MIAE表面拥有空间结构与苯丙氨酸对硝基苯胺的酰胺解反应过渡态构型互补的分子印迹空穴，该印迹空穴的存在赋予了MIAE优异的催化活性。催化性能测试结果表明，MIAE反应速率常数为非分子印迹聚合物的4倍，且该人工酶在重复使用5次后催化反应活性无明显下降。Guo等^［130］^以UiO-66为骨架，以有机膦神经毒剂（OP）为模板分子，制备了一种兼具MOF和分子印迹复合物特点的人工酶MOF@PHIPMIC。污染物降解测试结果表明，MOF@PHIPMIC可以催化纯水中的二甲基-4-硝基苯磷酸酯（DMNP）水解。且由于共聚过程中甲基丙烯酸甲酯的引入，MOF@PHIPMIC具有一定的pH缓冲性能。虽然现有纳米酶具有优异的稳定性和催化活性，但是仍不具有与天然酶相当的选择性。为获得与天然酶选择性相当的人工酶，Amine等^［131］^模仿天然酶的脱辅酶-辅酶系统，以分子印迹聚合物作为脱辅酶，纳米酶为辅酶，构筑了分子印迹人工酶体系。Amine等^［131］^将具有过氧化物催化活性的纳米酶Fe_3_O_4_-Lys-Cu与MIP相结合，制备了一种对L-DOPA和多巴胺具有优异催化活性的新型人工酶MIP-Fe_3_O_4_-Lys-Cu。催化性能测试结果表明，相较于Fe_3_O_4_-Lys-Cu，MIP-Fe_3_O_4_-Lys-Cu的催化活性提高了20倍。此外，这一构筑策略还可以显著提高分子印迹过程中模板分子脱除速度，大幅提高MIP的合成效率，使MIP-Fe_3_O_4_-Lys-Cu具有批量化生成潜力。

## 6 分子印迹技术在其他领域中的应用

除有机催化领域以外，MIT现已被广泛应用于传感器、吸附分离等新兴领域中。为检测环境中最常见的人体内分泌破坏物双酚A，Jana等^［132］^将聚丙烯酸酯、*β*-环糊精、氧化还原石墨烯与双酚A通过共价连接制备了一种具有电化学传感器应用潜力的MIP。该MIP的分子印迹空穴可以与*β*-环糊精共同与双酚A相互作用，实现双酚A的捕获，且对双酚A检出限较低，双酚A的线性浓度范围为0.02~1.0 ppm，检出限为8 ppb，满足实际水体中双酚A污染监测需求。Zhen等^［133］^通过硼亲和可控表面印迹策略，将模板分子固定在硼酸功能化基质表面，以具有优异生物相容性的物质为功能单体，在基质表面聚合后形成具有特定结构的3D分子印迹聚合物。该策略简化了分子印迹步骤，扩大了MIT应用范围，所得MIP现已广泛应用于疾病诊断、癌细胞靶向识别等生物识别场景。Pan和Li等^［134］^以医药中间体腺苷-5′-单磷酸为模板分子，5-（2-甲氧基乙烯基）-2′-脱氧尿苷和丙烯酰胺为功能单体，在戊二醛介导下在Fe_3_O_4_表面逐层印迹组装，制备了一种适用于复杂应用环境且可快速磁响应恢复的分子印迹吸附剂。吸附性能测试结果表明，该吸附剂饱和吸附量高达105.72 μmol/g，且磁响应性降低了该吸附剂回收难度，减少了回收损失。得益于分子印迹复合膜材料优异的分子识别性能，分子印迹膜已广泛应用于废水中有害物质的分离吸附。Liu和Qin等^［135］^通过分子印迹技术，以苯酚为模板分子，4-VPy为功能单体，EGDMA为交联剂，制备了一种对苯酚有选择性吸附的多层分子印迹复合膜材料m-MIM。分子印迹空穴的存在赋予了m-MIM极高的吸附容量和优异的分子识别能力，使m-MIM的苯酚吸附容量高达51.40 mg/g，且对苯酚的选择系数大于7.6，满足废水中苯酚的定向吸附分离处理需求。

## 7 总结与展望

分子印迹技术是一种具有特定识别位点的分子识别材料的制备方法，在催化领域中起到了重要作用。通过分子印迹技术，可以针对目标分子的特异性空间取向、特征官能团和化学反应途径，定制聚合物框架中分子印迹空穴空间结构和催化反应活性位点，最终获得具有酶催化特点、稳定性优于天然酶的MIC，还可对天然酶不适用的化学反应实现高效催化。此外，分子印迹技术可以大幅提高级联反应器中不同催化过程的分子识别精度和催化效率，在级联反应催化剂和智能反应器的制备中具有广阔应用前景。

现阶段，主要通过本体聚合、悬浮聚合、沉淀聚合和表面印迹等策略合成所需MIC。在制备过程中，通过调整印迹分子、功能单体（配体）和交联剂种类及比例，可以有效调控MIC的催化活性。表面印迹策略拓宽了分子印迹技术在有机催化领域中的应用范围，增加了模板分子的洗脱率，提高了MIC的制备效率，使MIC的批量化制备成为可能。

当前，分子印迹技术主要应用于催化水解反应、氧化反应、还原反应、偶联反应和聚合物反应器。在催化剂制备过程中利用分子印迹技术定制催化剂表面空穴形貌，赋予催化剂优秀的分子识别能力和催化反应活性，MIC目前还展现出优秀的产物择形催化能力，甚至可以高效合成手性产物。在催化体系的构筑过程中，印迹复合物的构型越稳定，分子印迹空穴的构型越明确，则越利于制备出具有优异分子识别能力和催化活性的MIC。在分子印迹过程中引入Co等过渡金属离子可以大幅提高印迹复合物的稳定性，还能在一定程度上提高MIC的催化活性。向分子印迹催化体系中引入Ru、Au、Ag等贵金属和Fe、Co、Ni等非贵金属催化活性位可以进一步提高催化剂的催化活性，现已在有机产物的制备和环境污染物的人工降解领域中展现出广阔的应用前景。为进一步提高MIC的催化活性、抗干扰能力和稳定性，以分子筛、MOF等稳定的无机多孔材料以及石墨烯等无机纳米材料为载体可以制备更加高效、稳定的MIC。此外，为实现级联反应产物的连续化高效制备、智能化制备，通过分子印迹技术制备聚合物反应器、智能反应器，引入分子印迹空穴，可以大幅减少不同反应之间的干扰，提高最终产物的生成效率。分子印迹技术可以赋予催化剂表面大量具有特定物理结构的印迹空穴，给予催化剂优异的分子识别能力。分子印迹技术拓展了催化剂应用场景，提供了新的催化剂制备思路。通过分子印迹技术，可以在具有催化活性或无催化活性的载体表面实现特定构型催化活性位点的装配，实现分子级催化活性单元的定制和组装。未来，根据特定的应用场景和产品类型，利用分子印迹技术，选用功能材料作为载体，可实现功能化催化剂的经济性制备。

然而，在MIC制备过程中，不同的聚合策略对MIC的内部微孔结构和骨架稳定性造成影响，最终影响MIC的催化活性。根据MIC的自身特性和应用场景，不同的制备方法还存在以下不足：（1）本体聚合策略中涉及耗时耗能的研磨、筛分工序，容易导致MIC内部微观结构破碎，所得催化剂颗粒尺寸不均一，MIC重复性能差，难以通过该策略实现MIC的批量制备；（2）交联聚合策略制备MIC过程中模板分子包埋过深，增大模板分子洗脱难度和催化剂中各元素定量控制难度。目前，大量先进的表征技术被广泛应用于MIC的结构鉴定和分析，但是现有表征技术还不能完全解析MIC的准确结构，主要表现如下：（1）利用ICP-AES、EA和TGA表征MIC结构的过程均对MIC造成不可逆的结构破坏，且三者之间不能有效衔接和对应；（2）利用SEM、TEM表征MIC的形貌时，只能获得MIC部分形貌信息，无法获得催化剂的整体形貌和结构信息。在MIC应用于多种催化反应方面，还存在一定局限性：（1）MIC虽然具有优于天然酶的热稳定性，但是无法应用于高温热催化体系；（2）MIC的专一性可有效催化某一特定化学反应，但无法连续催化连续/串联反应；（3）聚合物型MIC印迹空穴结构较为敏感，印迹复合物构型稳定性过强或过弱都会严重影响MIC的催化活性和分子识别能力。

本文根据对目前MIC在催化领域中应用的文献评述结果，对分子印迹技术在催化领域的未来发展提出以下建议：（1）在MIC制备过程中，根据MIC特性和应用环境，采用如光聚合法、沉淀聚合法等聚合策略交联聚合，以获得内部空穴微结构和骨架结构更加稳定的MIC，实现高性能MIC的制备。（2）拓宽分子印迹技术的应用范围，将分子印迹技术作为一种表面修饰工程的通用技术手段，实现按照特定需求改善材料的表面性质和物理化学性能，克服印迹复合物包埋过深的缺点，降低MIC内各元素定量控制难度，简化MIC制备流程。（3）在MIC元素表征过程中，利用X射线光电子能谱、俄歇电子能谱、色谱等对样品需求较少或对样品破坏程度较低的表征技术表征MIC元素含量和分布信息，以获得MIC的元素定性和定量数据。（4）在MIC形貌表征过程中，利用高分辨质谱、傅里叶变换红外光谱、紫外光谱、拉曼光谱、核磁共振波谱等先进表征技术获得MIC的碎片信息或化学成键信息，便于后续结构解析。（5）使用石墨烯、分子筛、氧化铝等无机多孔材料为载体制备MIC，进一步提高MIC的热稳定性，同时利用载体的多孔结构为催化剂提供更多的反应活性位点或反应通道，进一步提高MIC的催化活性。（6）利用分子印迹材料优异的分子识别能力和催化反应活性，将分子印迹技术应用于多功能催化剂（如多金属催化剂、级联反应器、智能反应器等）制备过程，以期获得可催化串联、耦合、竞争等反应的高活性高选择性MIC。（7）在分子印迹催化体系构筑过程中，针对不同应用场景的特点，以过渡金属离子为枢轴构建印迹复合物，通过控制功能单体结构与金属离子周围配体种类及数量，调控印迹复合物的构型稳定性，以获得催化性能优异的MIC。
